# Circular RNA TRAPPC6B inhibits intracellular *Mycobacterium tuberculosis* growth while inducing autophagy in macrophages by targeting microRNA‐874‐3p

**DOI:** 10.1002/cti2.1254

**Published:** 2021-02-18

**Authors:** Hou‐Long Luo, Jiang Pi, Jun‐Ai Zhang, En‐Zhuo Yang, Huan Xu, Hong Luo, Ling Shen, Ying Peng, Gan‐Bin Liu, Cai‐Mei Song, Ke‐Yue Li, Xian‐Jin Wu, Bi‐Ying Zheng, Hong‐Bo Shen, Zheng W Chen, Jun‐Fa Xu

**Affiliations:** ^1^ Department of Clinical Immunology Institute of Laboratory Medicine Guangdong Provincial Key Laboratory of Medical Molecular Diagnostics Guangdong Medical University Dongguan China; ^2^ Department of Microbiology and Immunology Center for Primate Biomedical Research University of Illinois College of Medicine Chicago IL USA; ^3^ Clinic and Research Center of Tuberculosis Shanghai Key Lab of Tuberculosis Shanghai Pulmonary Hospital Tongji University School of Medicine Shanghai China; ^4^ Department of Clinical Laboratory Huizhou Municipal Central Hospital Huizhou China; ^5^ Department of Respiration Dongguan 6th Hospital Dongguan China

**Keywords:** autophagy, circTRAPPC6B, macrophage, miR‐874‐3p, *Mycobacterium tuberculosis*

## Abstract

**Objectives:**

Genetic and epigenetic mechanisms regulate antimicrobial immunity against *Mycobacterium tuberculosis* (Mtb) infection.

**Methods:**

The present study assessed circular RNA TRAPPC6B (circTRAPPC6B) for antimicrobial immune functions and defined mechanisms wherein circTRAPPC6B regulates Mtb growth, autophagy and microRNA in macrophages.

**Results:**

The Mtb infection of monocytes/macrophages resulted in a significantly decreased level of circTRAPPC6B that inhibited intracellular Mtb growth in macrophages. Conversely, circTRAPPC6B expression enhanced autophagy or autophagy‐associated protein LC3‐II production in Mtb‐infected macrophages. circTRAPPC6B‐enhanced autophagy aggregation or sequestration was also observed in fluorescence *in situ* hybridisation (FISH) analysis and confocal imaging. Mechanistically, circTRAPPC6B targets an inhibiting element miR‐874‐3p, as shown by bioinformatics, dual‐luciferase reporter gene analysis and pull‐down assay, respectively. Notably, miR‐874‐3p prohibited autophagy via suppressing autophagy protein ATG16L1 by binding to its 3′‐untranslated region (UTR) in Mtb‐infected macrophages and thus promoting intracellular Mtb growth. Concurrently, circTRAPPC6B enhanced autophagy in Mtb‐infected macrophages by blocking the ability of miR‐874‐3p to inhibit ATG16L1. Thus, circTRAPPC6B antagonises the ability of *miR‐874‐3p* to suppress *ATG16L1* expression and activate and enhance autophagy sequestration to restrict Mtb growth in macrophages.

**Conclusion:**

The current findings suggested that both circTRAPPC6B and miR‐874‐3p mechanisms can be explored as potential therapeutics against Mtb infection.

## Graphical abstract

In this study, we found that circTRAPPC6B could inhibit intracellular *Mycobacterium tuberculosis* (Mtb) growth by inducing autophagy in Mtb‐infected macrophages through abolishing the suppression of miR‐874‐3p on ATG16L1 expression. This study highlights circTRAPPC6B as a potential therapeutic agent for tuberculosis control.

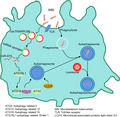

## Introduction

Tuberculosis (TB) is a transmissible, airborne disease caused by *Mycobacterium tuberculosis* (Mtb), with approximately 9 million new cases and 1.5 million deaths annually.^1^ Traditional anti‐TB chemotherapy has shown low efficacy in TB control, mainly due to the prevalence of virulent and multidrug‐resistant Mtb.^2^ Recently, host‐directed therapies that modulate the host immune responses have received considerable attention because it avoids the development of multidrug resistance.^3^, ^4^ Thus, an improved understanding of immune responses to Mtb infection is critical for identifying novel therapeutic targets for TB control.

Resident alveolar macrophages are present on the lung epithelia, forming the first line of defence against Mtb infection.^5^ Once inhaled, Mtb enters the alveolar macrophages via various phagocytic receptors and resides in the phagosomes that immediately mature to antimicrobial phagolysosomes. The delivery of Mtb into phagolysosomes relies on multiple trafficking pathways, including microtubule‐associated protein‐1 light chain 3 (LC3)‐associated phagocytosis and autophagy. During the lysosomal trafficking, Mtb activates various cellular processes, such as apoptosis, antigen presentation and autophagy, lethal to Mtb.^6^ However, Mtb can evade these cellular processes, resulting in the development of TB disease.^7^

Autophagy represents an intracellular process mediated by autophagosomes that transport cellular components to the lysosomes for degradation. Autophagosome formation requires the conversion of LC3‐I (unconjugated cytosolic form) to LC3‐II (autophagosomal membrane‐associated phosphatidylethanolamine‐conjugated form). Therefore, the amount of LC3‐II reflects the number of autophagosomes, serving as the most widely used autophagy marker.^8^, ^9^ Autophagy acts as an innate immunity with a crucial role in eliminating intracellular Mtb.^10^ The physiological activation of autophagy in macrophages facilitates mycobacterial phagosomes to mature into phagolysosomes, suppressing the intracellular survival of Mtb.^11^ In contrast, impaired autophagy increases Mtb survival and correlates with poor outcomes in patients with Mtb infection.^10^ During infection, the increased production of interferon‐gamma (IFN‐γ) activates macrophages to induce autophagy, facilitating the lysosomal degradation of Mtb by overcoming the Mtb‐imposed block of phagosome maturation.^12^ Many autophagy‐related proteins (ATGs), such as ATG5, ATG12 and ATG16L1, are involved in the innate immune clearance of Mtb. For example, ATG16L1 deficiency in macrophages protects Mtb from NADPH oxidase and phagocytosis.^13^ MicroRNA (miR)‐20a‐mediated silencing of ATG7 and ATG16L1 inhibits autophagy and promotes the survival of TB vaccine Bacillus Calmette‐Guérin (BCG) in macrophages.^14^ Therefore, the activation of autophagy in macrophages represents a promising therapeutic strategy in anti‐TB therapy.

Circular RNAs (circRNAs) are endogenous non‐coding RNAs characterised by a covalently closed loop structure lacking 5′ cap and 3′ poly‐A tail.^15^, ^16^, ^17^ Previous studies reported that circRNAs are involved in multiple biological functions, including autophagy, by sponging miRNAs.^15^, ^18^ For example, circHIPK2 regulates astrocyte activation via the cooperation of autophagy and ER stress by targeting miR‐124‐2HG.^19^ Intriguingly, miRNAs also play a critical role in autophagy by regulating host immunity during Mtb infection.^20^ A recent study demonstrated that Mtb infection upregulates the expression of miR‐23a‐5p in macrophages, promoting Mtb survival by suppressing autophagy through the Toll‐like receptor 2 (TLR2) signalling.^21^ However, little is known about the interaction between circRNAs and miRNAs in macrophage autophagy during Mtb infection.

Our previous study revealed that the peripheral blood mononuclear cells (PBMCs) of patients with active TB have significantly decreased expression of circTRAPPC6B as compared to those of healthy controls,^22^ suggesting that circTRAPPC6B might play an unfavorable role in Mtb survival. This study further investigated the role of circTRAPPC6B and its interaction with miRNAs in Mtb‐infected macrophages. The findings suggested that circTRAPPC6B targets miR‐874‐3p to abolish its suppression on ATG16L1, thereby inducing autophagy and inhibiting Mtb growth in macrophages.

## Results

### CircTRAPPC6B is significantly downregulated in PBMCs of patients with active TB and monocytes/macrophages with Mtb infection

**Figure 1:**
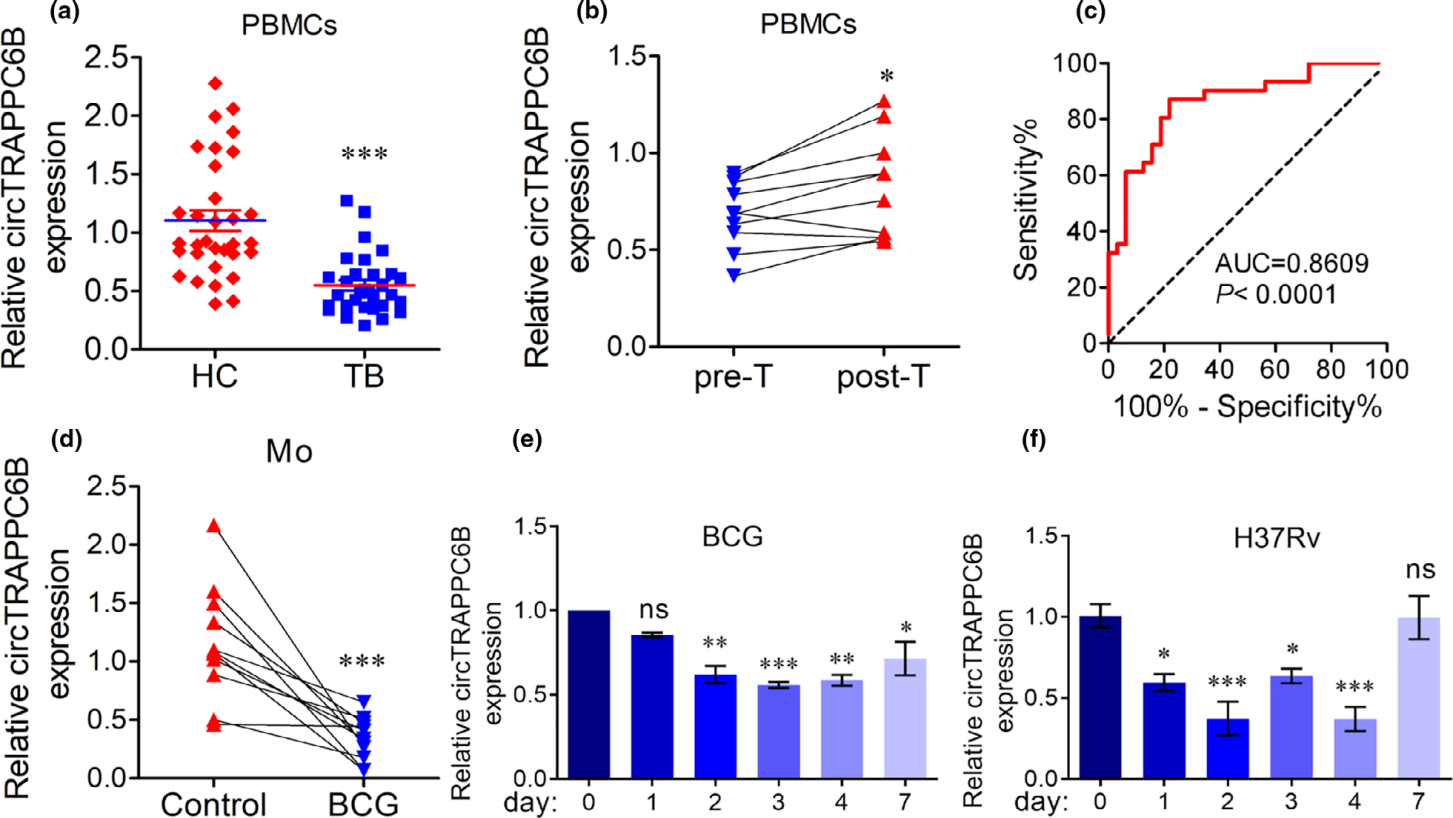
circTRAPPC6B was significantly downregulated in PBMCs from patients with active TB and Mtb‐infected monocytes and macrophages. **(a)** After PBMCs were freshly isolated from blood by standard Ficoll density gradient centrifugation, circTRAPPC6B expression was analysed in human PBMCs from patients with 32 active TB and 31 HC by qRT‐PCR. GAPDH was used as a housekeeping gene for normalising changes in circRNA gene expression. Data are expressed as mean ± SEM. ***P < 0.001 *vs* HC. **(b)** After 10 patients with active TB received individualised anti‐TB treatments, including isoniazid, rifampicin, pyrazinamide and ethambutol, circTRAPPC6B expression was analysed in PBMCs from 10 patients with active TB before (pre‐T) and after (post‐T) 1 month of anti‐TB therapy. Data are expressed as mean ± SEM. *P < 0.05 *vs* Pre‐T. **(c)** To evaluate the diagnostic value in active TB, circTRAPPC6B expression was analysed by the ROC curve. **(d)** After human peripheral monocytes were sorted from PBMCs by immunomagnetic positive selection, circTRAPPC6B expression was analysed in human peripheral monocytes with or without TB vaccine BCG infection at MOI = 10 for 24 h. Data are expressed as mean ± SEM. ***P < 0.001 *vs* uninfected control. **(e, f)** After THP‐1 macrophages were infected with BCG **(e)** at MOI = 10 and H37Rv **(f)** at MOI = 1 at different time points (0, 1, 2, 3, 4 and 7 days), circTRAPPC6B expression was analysed by qRT‐PCR. The data were obtained from three independent experiments. *P < 0.05, **P < 0.01, ***P < 0.001 *vs* day 0. BCG, Bacillus Calmette‐Guérin; HC, health control; MOI, multiplicity of infection; ns, no significance; PBMCs, peripheral blood mononuclear cells; qRT‐PCR, quantitative real‐time polymerase chain reaction; ROC, reactive operating characteristic curve; TB, tuberculosis.

In a previous study, we reported that PBMCs in patients with active TB had significantly downregulated circTRAPPC6B expression compared to healthy controls.^22^ In the present study, we further confirmed the weak expression of circTRAPPC6B in PBMCs of 32 enrolled patients with active TB and 31 healthy volunteers (Figure 1a). One month of standardised anti‐TB treatment rescues the expression of circTRAPPC6B in PBMCs (Figure 1b), suggesting an unfavorable role of circTRAPPC6B in Mtb growth. In addition, the area under the curve (AUC) of circTRAPPC6B in diagnosing active TB was 0.8609 (*P* < 0.0001; Figure 1c), indicating that circTRAPPC6B serves as a diagnostic marker for active TB. These results indicated that circTRAPPC6B was downregulated due to Mtb infection.

To confirm these conclusions in both *ex vivo* monocytes/macrophages and *in vitro* macrophage cell lines, we analysed the expression of *circTRAPPC6B* in Mtb‐infected human PBMC and THP‐1 macrophages. Also, due to the limited BSL‐3 lab (P3) availability, we used BCG as a model of Mtb to explore the potential mechanism of *circTRAPPC6B* involved in TB and then further confirmed the results by Mtb (H37Rv)‐infected macrophages. Because monocytes and alveolar macrophages constitute the first line of defence against TB, we detected the expression of circTRAPPC6B in BCG‐infected human monocytes and macrophages. The data from real‐time quantitative polymerase chain reaction (qRT‐PCR) showed that BCG‐infected human PBMCs exhibited significantly attenuated expression of circTRAPPC6B compared to the uninfected cells (Figure 1d). Significantly attenuated expression of circTRAPPC6B was also observed in BCG‐infected THP‐1 macrophages (Figure 1e), which was also confirmed in H37Rv‐infected THP‐1 macrophages (Figure 1f). These results further confirmed our previous findings that weak expression of circTRAPPC6B was involved in Mtb infection.

### CircTRAPPC6B is mainly located in the cytoplasm of macrophages

**Figure 2:**
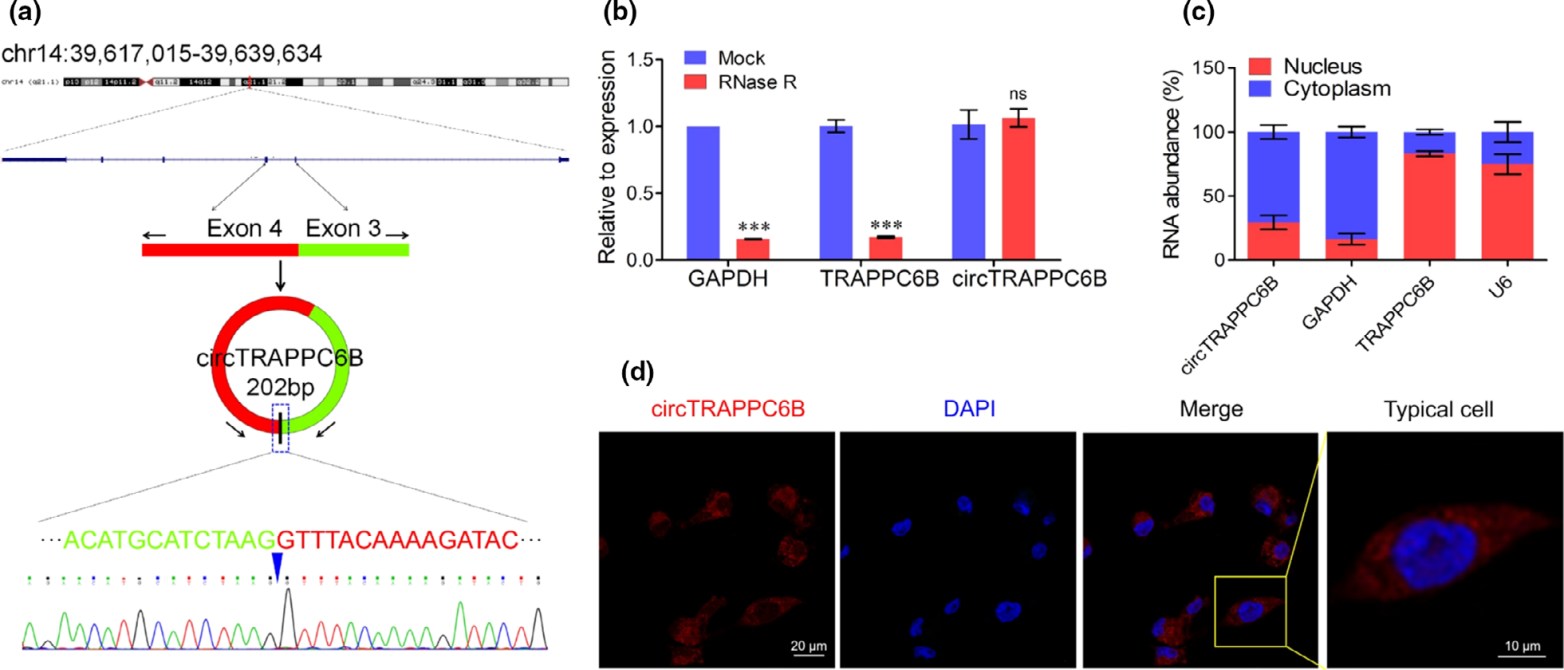
Identification of the circular structure and cellular distribution of circTRAPPC6B. **(a)** Schematic illustration displays that circTRAPPC6B is located at chromosome 14q21.1. The precise genomic location is 39,617,015–39,639,634. circTRAPPC6B is cyclised from exons 3 and 4 of TRAPPC6B. The PCR products of circTRAPPC6B were evaluated by Sanger sequencing. **(b)** After treatment with or without (Mock) RNase R according to the protocol of manufacturer, the relative expression of circTRAPPC6B and linear mRNAs of GAPDH and TRAPPC6B was analysed in THP‐1 cells by qRT‐PCR. The data were obtained from three independent experiments. Data are expressed as mean ± SEM. ***P < 0.001 *vs* Mock. **(c)** After treatment with Nuclear and Cytoplasmic Extraction Reagents according to the protocol of manufacturer, circTRAPPC6B expression was analysed in the subcellular distribution of THP‐1 cells infected with BCG for 24 h by qRT‐PCR. The data were obtained from three independent experiments. **(d)** After infection with BCG at MOI = 10 for 24 h, THP‐1 macrophages were collected and incubated with biotin‐conjugated circTRAPPC6B probe overnight at 37°C. Representative immunofluorescence confocal image showing the subcellular distribution of circTRAPPC6B. Scale bar, 20 μm. Scale bar of typical cell, 10 μm. PCR, polymerase chain reaction; qRT‐PCR, quantitative real‐time polymerase chain reaction; BCG, Bacillus Calmette‐Guérin; MOI, multiplicity of infection; FISH, fluorescence *in situ* hybridisation.

Next, we explored the generation and cellular location of circTRAPPC6B in macrophages. Bioinformatics revealed that circTRAPPC6B was derived from the back‐splicing of exons 3 and 4 of the TRAPPC6B gene (circBase ID: hsa_circ_0005836) located at chromosome 14q21.1; the precise genomic location was 39,617,015–39,639,634. The length of mature circTRAPPC6B was 202 bp, according to the circBase database (http://www.circbase.org/) (Figure 2a). In order to exclude the possibility that the qRT‐PCR products might originate from genomic rearrangements and trans‐splicing, we treated the RNAs with RNAse R before PCR. We observed that RNAse R digestion dramatically reduces the production of linear TRAPPC6B and GAPDH, but not that of circTRAPPC6B (Figure 2b), indicating that the splicing product was circular. Sanger sequencing further confirmed that the sequence of circTRAPPC6B produced by qRT‐PCR matched that in the circBase database (Figure 2a). Then, the cytoplasm and nucleus components of macrophages were extracted, and the expression of circTRAPPC6B was analysed, which indicated that circTRAPPC6B was mainly localised in the cytoplasm (Figure 2c). The FISH assay also confirmed the location of circTRAPPC6B in the cytoplasm of macrophages (Figure 2d, enlarged microscopy images are presented in Supplementary figure 1). These data collectively suggest that circTRAPPC6B, with a circular structure, is predominantly localised in the cytoplasm of macrophages.

### CircTRAPPC6B inhibits intracellular Mtb growth in macrophages

**Figure 3:**
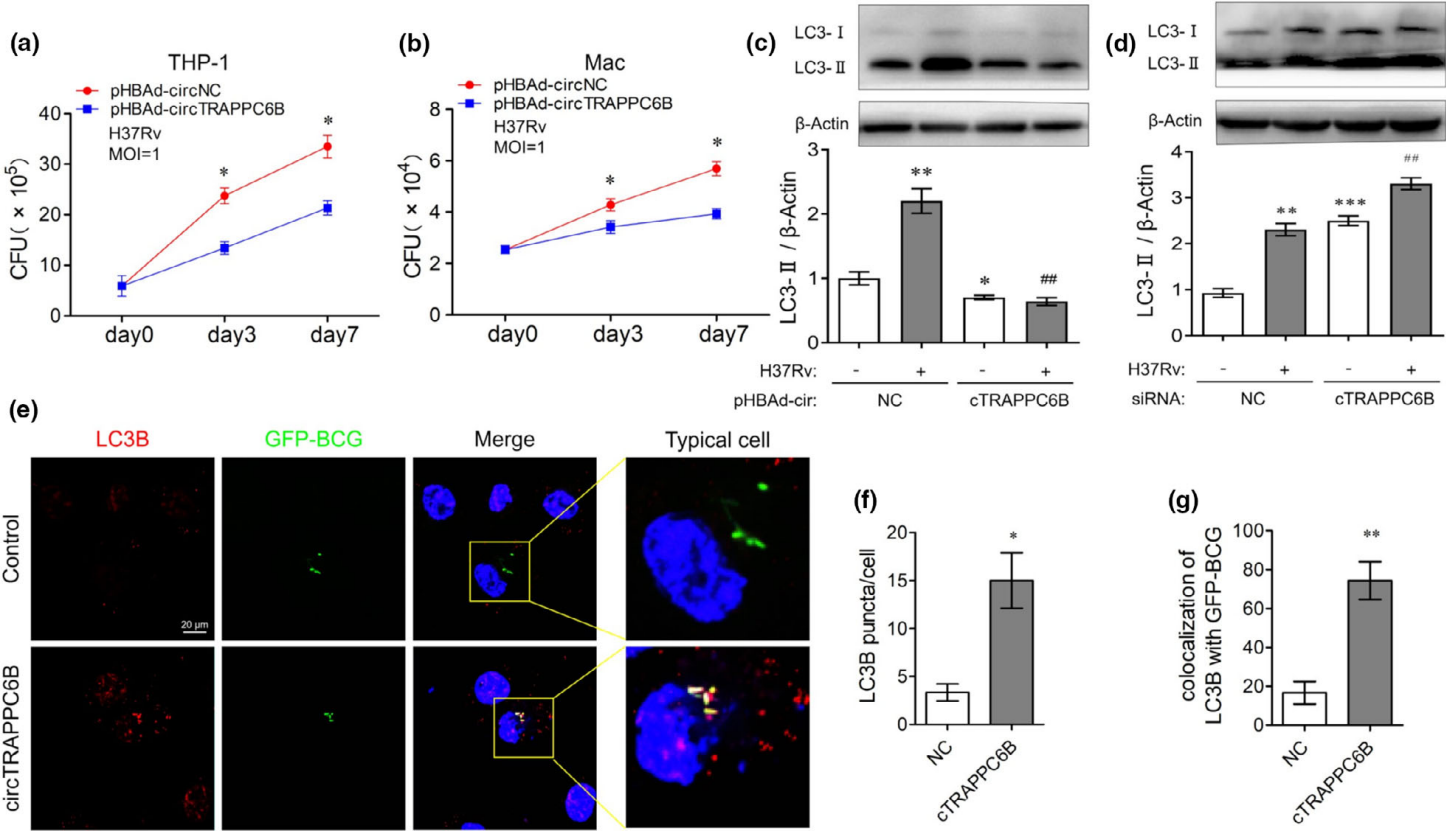
Overexpression of circTRAPPC6B inhibited intracellular Mtb growth while activating autophagy in Mtb‐infected macrophages. **(a, b)** After transfection with circTRAPPC6B overexpressing plasmids or control plasmids for 24 h, THP‐1 macrophages **(a)** and macaque spleen macrophage **(b)** were infected with *Mycobacterium* H37Rv at MOI = 1 for 3 or 7 days. The growth of the bacilli was evaluated by CFU count. The data were obtained from six independent experiments. Data are expressed as mean ± SEM. *P < 0.05 *vs* day 0 (*n* = 6). **(c, d)** After transfection with siRNA against circTRAPPC6B **(c)** or plasmids overexpressing circTRAPPC6B **(d)** for 24 h, THP‐1 macrophages were infected with *Mycobacterium* H37Rv at MOI = 1 for 24 h. Representative Western blot showing the change of the LC3‐I and LC3‐II protein expression in THP‐1 macrophages. The data were obtained from three independent experiments. Data are expressed as mean ± SEM. *P < 0.05, **P < 0.01, ***P < 0.001 *vs* uninfected and untransfected control. ^##^ *P* < 0.01 *vs* H37Rv‐infected but untransfected control. **(e)** After transfection with circTRAPPC6B overexpressing vectors or control vectors (NC) for 24 h, THP‐1 macrophages were infected with GFP‐BCG at MOI = 10 for 24 h. THP‐1 macrophages were incubated with anti‐LC3 for 2h at room temperature and then fluorescently labelled secondary Ab for 1 h at room temperature. Representative immunofluorescence confocal image of THP‐1 macrophages showing the change of LC3B puncta and GFP‐BCG colocalisation. Scale bar, 20 μm. **(f, g)** Quantification assay of e. 100 cells were count in every independent experiment. The data were obtained from the mean number of LC3B puncta or colocalisation of LC3B with GFP‐BCG of three independent experiments. Data are expressed as mean ± SEM. *P < 0.05, **P < 0.01 *vs* NC (*n* = 3). MOI, multiplicity of infection; CFU, colony‐forming unit; GFP, green fluorescence protein; BCG, Bacillus Calmette‐Guérin.

To explore the roles of circTRAPPC6B involved in Mtb infection, we transfected the THP‐1 macrophages with overexpression plasmid pHBAd‐circTRAPPC6B (pHBAd‐cir) with high transfection efficiency (Supplementary figure 2). Also, compared with pHBAd‐control vector (vector), pHBAd‐cir transfected cells demonstrated significant increase of circTRAPPC6B expression (Supplementary figure 3). To explore the role of circTRAPPC6B in intracellular Mtb growth, we performed a colony formation unit (CFU) assay in H37Rv‐infected macrophages, of which the H37Rv infection was performed at a multiplicity of infection (MOI) of 1. At 4 h after infection, extracellular non‐internalised bacilli were removed, and this time point was recorded as day 0. The results showed that after H37Rv infection, THP‐1 macrophages overexpressing circTRAPPC6B had significantly decreased CFU compared to control cells at days 3 and 7, respectively (Figure 3a). To further confirm these conclusions by tissue primary macrophages, we tested the effects of circTRAPPC6B on H37Rv growth in H37Rv infected macaque spleen primary macrophages. Similar results were observed in the primary macrophages in the macaque spleen (Figure 3b). These findings suggested that the overexpression of circTRAPPC6B suppresses the intracellular Mtb growth in macrophages.

### CircTRAPPC6B activates autophagy in Mtb‐infected macrophages

**Figure 4:**
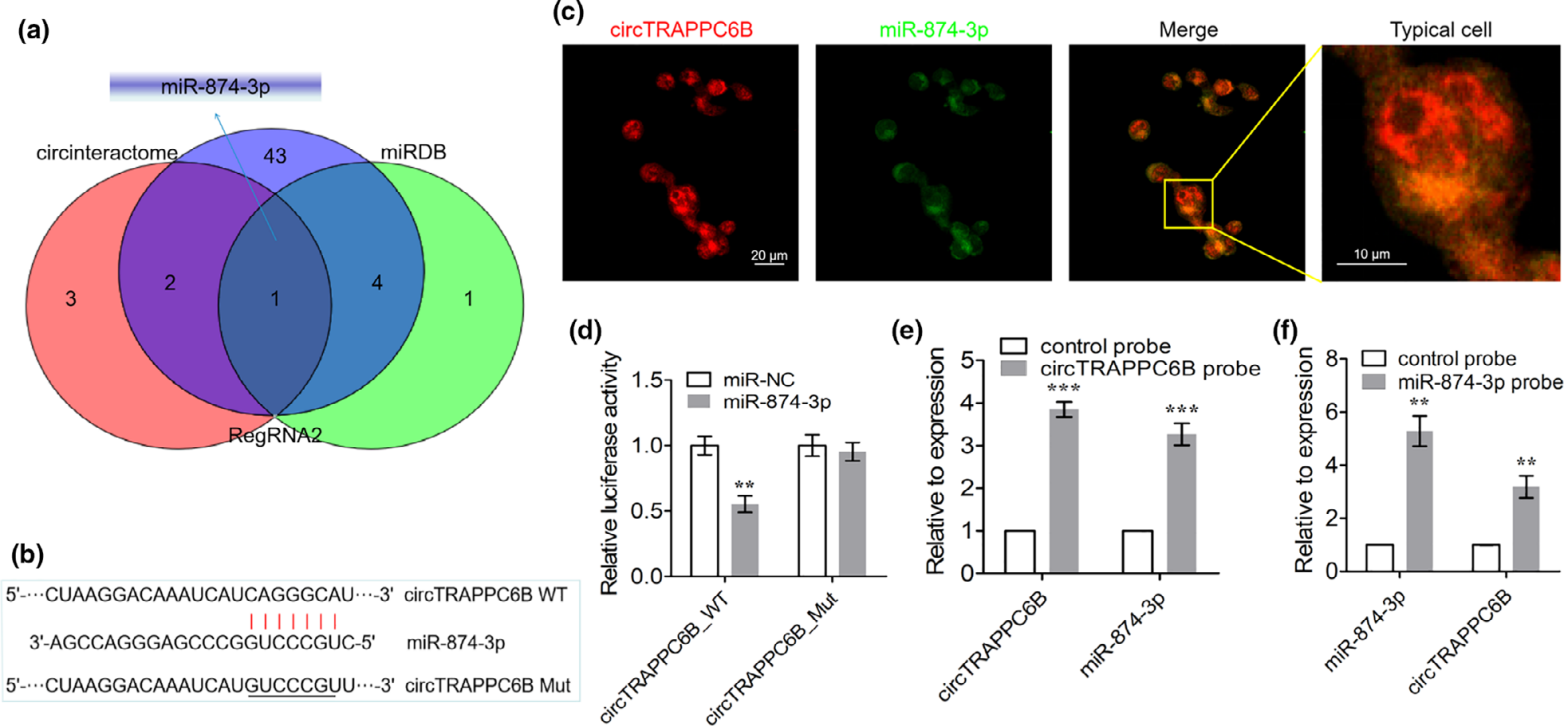
miR‐874‐3p was identified as the target of circTRAPPC6B. **(a)** The intersection of CircInteractome, miRDB and RegRNA 2.0 databases was analysed to predict potential miRNA targets of circTRAPPC6B. **(b)** A schematic diagram showing the predicted binding site between 3’UTR of circTRAPPC6B and miR‐874‐3p. Wide‐ and mutant‐type circTRAPPC6B 3’UTR luciferase reporter vectors were constructed and cotransfected with miR‐874‐3p mimic in HEK293T cells. **(c)** After infection with BCG at MOI = 10 for 24 h, THP‐1 macrophages were collected and incubated with biotin‐conjugated circTRAPPC6B and digoxin‐conjugated miR‐874 probes overnight at 37°C. Representative immunofluorescence confocal image showing partly colocalisation of circTRAPPC6B with miR‐874‐3p in the cytoplasm of THP‐1 macrophages. Scale bar, 20 μm. Scale bar of typical cell, 10 μm. **(d)** The relative luciferase activity was determined at 48 h after cotransfection with circTRAPPC6B harbouring wild‐type or mutant miR‐874‐3p binding site and miR‐874‐3p mimics or negative control (NC) in HEK293T cells. The data were obtained from three independent experiments. Data are expressed as mean ± SEM. **P < 0.01 *vs* NC. **(e)** CircRNA pull‐down assay was performed in THP‐1 macrophages using biotin‐conjugated circTRAPPC6B to assess the interaction between circTRAPPC6B and miR‐874‐3p, followed by qRT‐PCR to detect circTRAPPC6B and miR‐874‐3p enrichment. The data were obtained from three independent experiments. Data are expressed as mean ± SEM. ***P < 0.001 *vs* control probe. **(f)** miRNA pull‐down assay was performed in THP‐1 macrophages using digoxin‐conjugated miR‐874 probes, followed by qRT‐PCR to detect miR‐874‐3p and circTRAPPC6B enrichment. The data were obtained from three independent experiments. Data are expressed as mean ± SEM. **P < 0.01, ***P < 0.001 *vs* control probe. FISH, fluorescence *in situ* hybridisation.

Considering the critical roles of autophagy in eliminating intracellular Mtb,^10^, ^11^, ^12^ we sought to investigate whether circTRAPPC6B affects autophagy in Mtb‐infected macrophages. As a marker of autophagy, the expression of LC3‐II was detected by Western blot. We found that the knockdown of circTRAPPC6B by siRNA transfection significantly attenuated LC3‐II expression, whereas the overexpression of circTRAPPC6B by plasmid transfection remarkably enhanced the expression of LC3‐II protein in BCG‐infected THP‐1 macrophages (Supplementary figure 4). Importantly, the knockdown of circTRAPPC6B partially but significantly reversed BCG infection‐induced LC3‐II upregulation in THP‐1 macrophages (Supplementary figure 4a). Conversely, the overexpression of circTRAPPC6B further enhanced LC3‐II upregulation in BCG‐infected cells (Supplementary figure 4b). Similar results were observed in H37Rv‐infected THP‐1 macrophages (Figure 4c and d), which further suggested the potential roles of circTRAPPC6B in regulating autophagy of Mtb‐infected macrophages. Consistently, FISH assay was performed using biotin or digoxin‐conjugated probes to detect the intracellular colocalisation. Herein, we demonstrated that the overexpression of circTRAPPC6B resulted in the dramatically increased number of LC3B‐positive puncta (red) in BCG‐infected THP‐1 cells compared to control vectors (Figure 3e and f, enlarged microscopy images are presented in Supplementary figure 5). The overexpression of circTRAPPC6B also enhanced the colocalisation (yellow) of LC3B puncta and GFP‐expressing BCG in THP‐1 macrophages (Figure 3e and g, enlarged microscopy images are presented in Supplementary figure 5). Taken together, these data suggest that circTRAPPC6B activates autophagy in Mtb‐infected macrophages.

### CircTRAPPC6B specifically targets miR‐874‐3p

circRNAs can specifically bind to miRNAs to counter miRNA‐mediated gene silencing. To investigate the mechanism underlying the role of circTRAPPC6B in Mtb growth and autophagy in macrophages, we predicted its potential miRNA targets using public databases CircInteractome, miRDB and RegRNA 2.0. The intersection of these databases revealed that circTRAPPC6B harboured a binding site for miR‐874‐3p (Figure 4a and b). Then, biotin‐conjugated circTRAPPC6B and digoxin‐conjugated miR‐874 probes were synthesised, hybridised with the cells, and incubated with anti‐biotin CY3‐conjugated or anti‐digoxin FITC‐conjugated secondary antibody to visualise the colocalisation by RNA FISH assay. Subsequently, we observed that circTRAPPC6B was colocalised with miR‐874‐3p in the cytoplasm of THP‐1 cells (Figure 4c, enlarged microscopy images are presented in Supplementary figure 6), which suggested that circTRAPPC6B could bind with miR‐874 in the cytoplasm of macrophages. Dual‐luciferase reporter gene assay demonstrated that the overexpression of wild‐type circTRAPPC6B reduced the luciferase activity of miR‐874‐3p mimics, whereas circTRAPPC6B with a mutated miR‐874‐3p binding site could not (Figure 4d). In addition, the biotin‐coupled RNA complex was used in the circRNA and miRNA pull‐down assay. We demonstrated that either circTRAPPC6B probe or miR‐874‐3p probe could pull down significantly enriched miR‐874‐3p and circTRAPPC6B as compared to the control probe (Figure 4e and f). Taken together, these findings indicated that circTRAPPC6B physically binds to miR‐874‐3p, acting as a miR‐874‐3p sponge.

### MiR‐874‐3p promotes intracellular Mtb growth while inhibiting autophagy in Mtb‐infected macrophages

**Figure 5:**
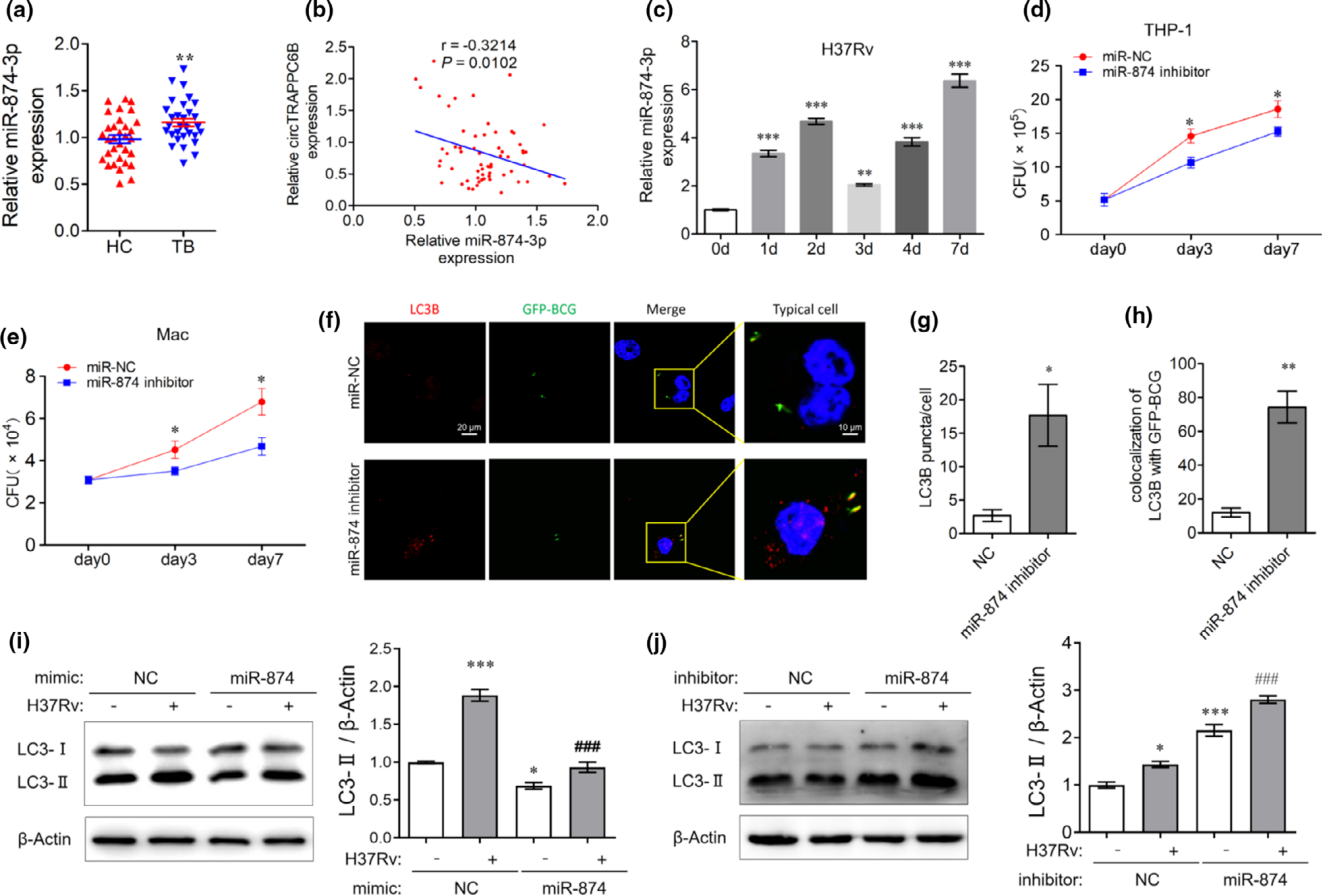
miR‐874‐3p regulated intracellular Mtb growth and autophagy in Mtb‐infected macrophages. **(a)** After PBMCs were freshly isolated from blood by standard Ficoll density gradient centrifugation, miR‐874‐3p expression was analysed in human PBMCs from patients with 32 active TB and 31 HC by qRT‐PCR. U6 was used as a housekeeping gene for normalising changes in miRNA gene expression. Data are expressed as mean ± SEM. **P < 0.01 *vs* HC. **(b)** Spearman’s correlation analysis between miR‐874‐3p and circTRAPPC6B expression in PBMCs of 32 TB patients. **(c)** After THP‐1 macrophages were infected with H37Rv at MOI = 1 at different time points (0, 1, 2, 3, 4, and 7 days), miR‐874‐3p expression was analysed by qRT‐PCR. The data were obtained from three independent experiments. Data are expressed as mean ± SEM. **P < 0.01, ***P < 0.001 *vs* 0 d. **(d, e)** After transfection with miR‐874‐3p inhibitor or negative control (miR‐NC) for 24 h, THP‐1 macrophages **(d)** and macaque spleen macrophage **(e)** were infected with *Mycobacterium* H37Rv at MOI = 1 for 3 or 7 days. The growth of the bacilli was evaluated by CFU count. The data were obtained from six independent experiments. Data are expressed as mean ± SEM. *P < 0.05 *vs* day 0 (*n* = 6). **(f)** After transfection with miR‐874‐3p inhibitor or negative control (miR‐NC) for 24 h, THP‐1 macrophages were infected with GFP‐BCG at MOI = 10 for 24 h. Then, THP‐1 macrophages were incubated with anti‐LC3B for 2h at room temperature and then fluorescently labelled secondary Ab for 1h at room temperature. Representative immunofluorescence confocal image of THP‐1 macrophages showing the change of LC3B puncta and GFP‐BCG colocalisation. Scale bar, 20 μm. **(g, h)** Quantification assay of f. 100 cells were count in every independent experiment. The data were obtained from the mean number of LC3B puncta or colocalisation of LC3B with GFP‐BCG of three independent experiments. Data are expressed as mean ± SEM. *P < 0.05, **P < 0.01 *vs* miR‐NC. **(i, j)** After transfection with miR‐874‐3p mimics **(i)** or inhibitor **(j)** for 24 h, THP‐1 macrophages were infected with *Mycobacterium* H37Rv at MOI = 1 for 24 h. Representative Western blot showing the change of the LC3‐I and LC3‐II protein expression in THP‐1 macrophages. The data were obtained from three independent experiments. Data are expressed as mean ± SEM. *P < 0.05, ***P < 0.001 *vs* uninfected and untransfected control; ^###^ *P* < 0.001 *vs* H37Rv‐infected but untransfected cells. qRT‐PCR, quantitative real‐time polymerase chain reaction; PBMCs, peripheral blood mononuclear cells; TB, tuberculosis; HC, health control; MOI, multiplicity of infection; CFU, colony forming unit; GFP, green fluorescence protein; BCG, Bacillus Calmette‐Guérin.

A recent study showed that miR‐874‐3p could inhibit autophagy by reducing LC3‐II expression in HeLa cells^23^; however, whether miR‐874‐3p affects Mtb growth and autophagy in Mtb‐infected macrophages remains to be explored. In contrast to the circTRAPPC6B expression, we found that PBMCs from patients with active TB had significantly increased miR‐874‐3p level compared to the respective controls (Figure 5a). Moreover, Spearman’s analysis showed a significant negative correlation between circTRAPPC6B and miR‐874‐3p expression in PBMCs (Figure 5b). BCG infection also induced significantly increase of miR‐874‐3p level in THP‐1 macrophages (Supplementary figure 7). In addition, significant increase of miR‐874‐3p level was also found in H37Rv‐infected THP‐1 macrophages (Figure 5c). These findings suggested that miR‐874‐3p might play opposite roles to circTRAPPC6B in TB.

Strikingly, the results of the CFU assay showed that miR‐874‐3p inhibitor significantly suppressed the intracellular H37Rv growth in both THP‐1 and macaque spleen macrophages (Figure 5d and e), indicating an essential role of miR‐874‐3p in promoting Mtb growth in macrophages. In addition, miR‐874‐3p inhibition resulted in significantly elevated number of LC3B‐positive puncta and enhanced colocalisation of LC3B and GFP‐BCG in GFP‐BCG‐infected THP‐1 macrophages (Figure 5f–h, enlarged microscopy images are presented in Supplementary figure 8). miR‐874‐3p mimics dramatically suppressed, whereas miR‐874‐3p inhibitor facilitated the conversion of LC3‐I to LC3‐II in THP‐1 macrophages irrespective of BCG (Supplementary figure 9a and b) and H37Rv infection (Figure 5i and j), suggesting that miR‐874‐3p is also essential for suppressing autophagy in macrophages. These data collectively suggested that miR‐874‐3p plays opposite roles to circTRAPPC6B in TB by promoting Mtb growth while inhibiting autophagy in macrophages.

### CircTRAPPC6B regulates autophagy in Mtb‐infected macrophages, possibly via miR‐874‐3p/ATG16L1

**Figure 6:**
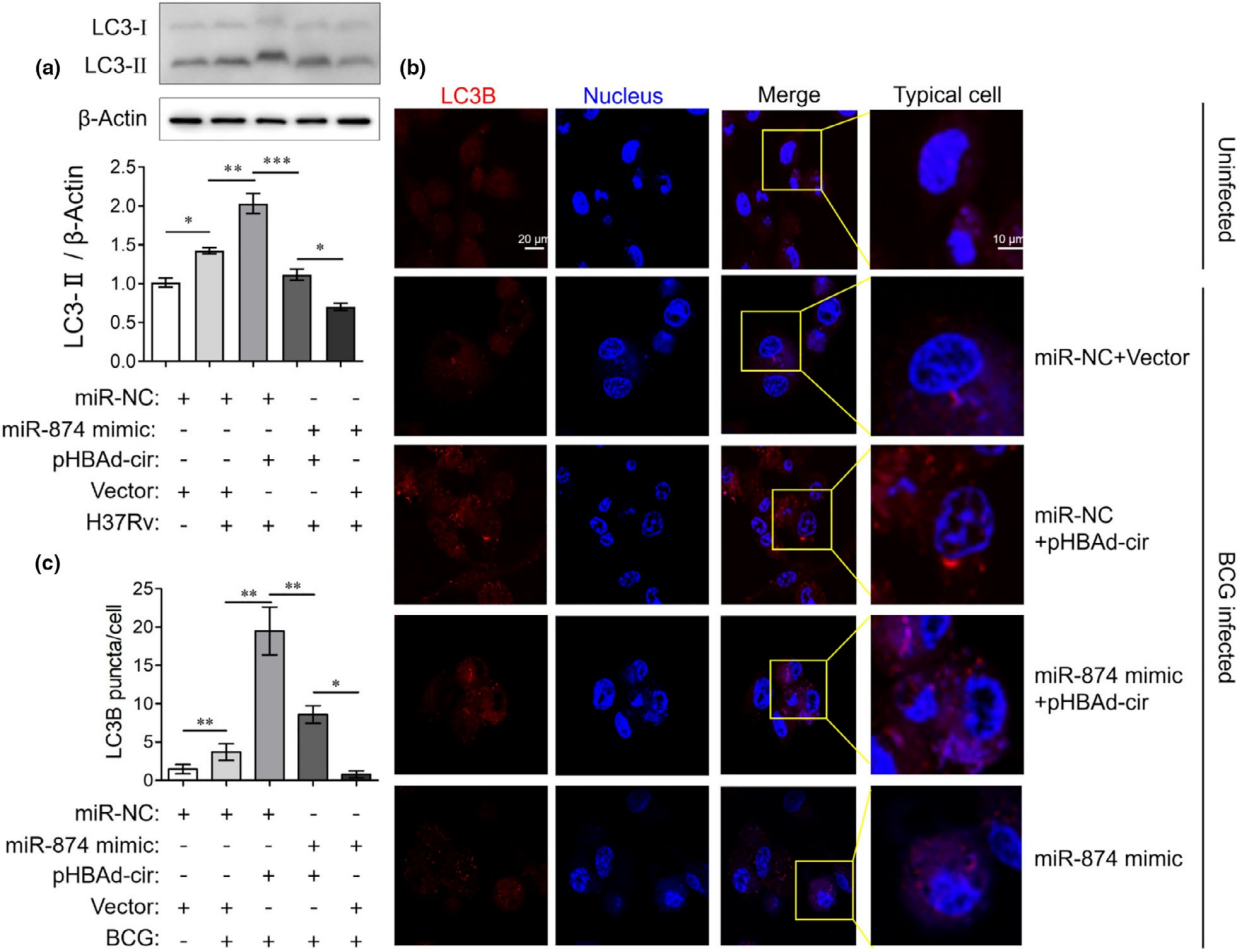
circTRAPPC6B regulated autophagy in Mtb‐infected macrophages via miR‐874‐3p. After transfection with circTRAPPC6B‐overexpressing vectors (pHBAd‐cir), miR‐874‐3p mimics, or corresponding negative controls as indicated for 24 h, THP‐1 macrophages were infected with BCG at MOI = 10 or *Mycobacterium* H37Rv at MOI = 1 for 24 h. **(a)** Representative Western blot showing the change of the LC3‐I and LC3‐II protein expression in H37Rv‐infected THP‐1 macrophages. The data were obtained from three independent experiments. Data are expressed as mean ± SEM. *P < 0.05, **P < 0.01 and ***P < 0.001. **(b)** THP‐1 macrophages were incubated with anti‐LC3B for 2h at room temperature and then fluorescently labelled secondary Ab for 1h at room temperature. Representative immunofluorescence confocal image of BCG‐infected THP‐1 macrophages showing the change of LC3B puncta. **(c)** Quantification of b. 100 cells were count in every independent experiment. The data were obtained from the mean number of LC3B puncta of three independent experiments. Data are expressed as mean ± SEM. *P < 0.05, **P < 0.01. BCG, Bacillus Calmette‐Guérin; MOI, multiplicity of infection.

Next, we sought to investigate whether miR‐874‐3p mediates the regulatory role of circTRAPPC6B in macrophage autophagy during Mtb infection. We found that miR‐874‐3p mimics reverse the LC3‐II conversion induced by circTRAPPC6B overexpression in BCG‐infected (Supplementary figure 10) and H37Rv‐infected THP‐1 macrophages (Figure 6a). Consistent results were observed in immunofluorescence assay obtained in BCG‐infected THP‐1 macrophages (Figure 6b and c, enlarged microscopy images are presented in Supplementary figure 11). These findings suggested that miR‐874‐3p could block the inductive role of circTRAPPC6B in macrophage autophagy during Mtb infection.

**Figure 7:**
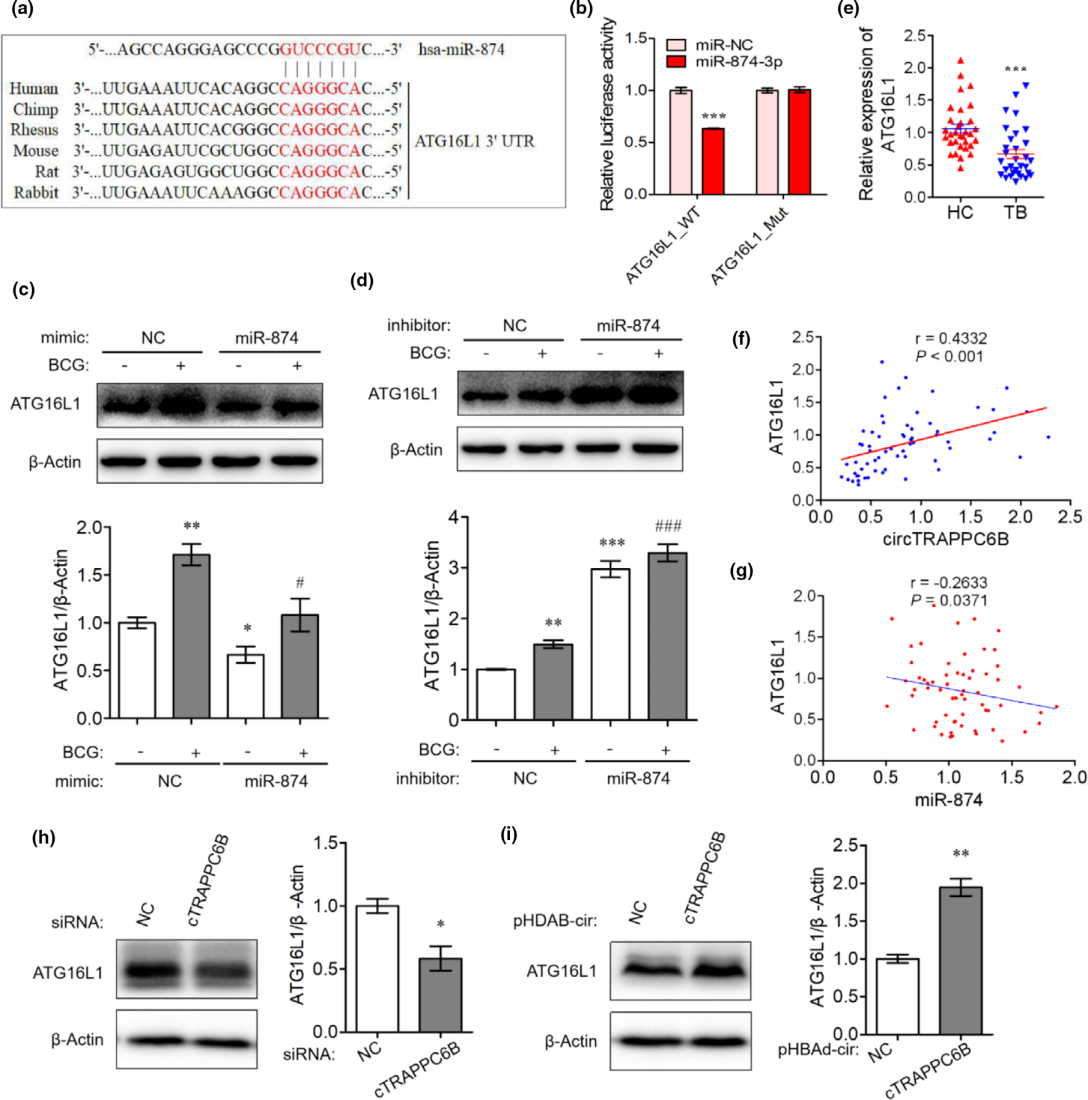
CircTRAPPC6B regulated autophagy in Mtb‐infected macrophage via miR‐874‐3p/ATG16L1. **(a)** A schematic diagram showing the predicted binding site between 3′UTR of ATG16L1 and miR‐874‐3p. Wide‐ and mutant‐type circTRAPPC6B 3’UTR luciferase reporter vectors were constructed. **(b)** After cotransfection with wild‐type or mutant ATG16L1 3′‐UTR expressing vectors and miR‐874‐3p mimics in HEK293T cells for 48h, dual‐luciferase reporter assay was performed to evaluate the binding between miR‐874‐3p and ATG16L1. The data were obtained from three independent experiments. Data are expressed as mean ± SEM. ***P < 0.001 *vs* miR‐NC. **(c, d)** After transfection with miR‐874‐3p mimics **(c)** or inhibitor **(d)** for 24 h, THP‐1 macrophages were infected with BCG at MOI = 10 for 24 h. Representative Western blot showing the change of the ATG16L1 protein expression in THP‐1 macrophages. The data were obtained from three independent experiments. Data are expressed as mean ± SEM. *P < 0.05, **P < 0.01, ***P < 0.001 *vs* uninfected and untransfected cells; ^#^ *P* < 0.05, ^###^ *P* < 0.001 *vs* BCG‐infected but untransfected cells. **(e)** After PBMCs were freshly isolated from blood by standard Ficoll density gradient centrifugation, ATG16L1 mRNA expression was analysed in human PBMCs from patients with 32 active TB and 31 HC by qRT‐PCR. GAPDH was used as a housekeeping gene for normalising changes in mRNA gene expression. Data are expressed as mean ± SEM. ***P < 0.001 *vs* HC. **(f)** *Spearman’s* correlation analysis between ATG16L1 and circTRAPPC6B expression in PBMCs of 32 TB patients. **(g)** *Spearman’s* correlation analysis between miR‐874‐3p and ATG16L1 expression in PBMCs of 32 TB patients. **(h‐i)** After transfection with siRNA against circTRAPPC6B **(h)** or plasmids overexpressing circTRAPPC6B **(i)** for 24 h, THP‐1 macrophages were infected with BCG at MOI = 10 for 24 h. Representative Western blot showing the change of the ATG16L1 protein expression in THP‐1 macrophages. The data were obtained from three independent experiments. Data are expressed as mean ± SEM. *P < 0.05 and **P < 0.01, *vs* uninfected or untransfected control. ATG16L1, Autophagy Related Protein 16 Like Protein 1; BCG, Bacillus Calmette‐Guérin; HC, health control; MOI, multiplicity of infection; PBMCs, peripheral blood mononuclear cells; TB, tuberculosis; UTR, untranslated regions.

To further investigate the mechanism underlying the role of miR‐874‐3p in macrophage autophagy, we conducted a bioinformatics analysis to identify its potential targets using TargetScan, miRanda and miRDB databases. It was found that the 3′‐UTR of ATG16L1 mRNA harboured a potential miR‐874‐3p binding site (Figure 7a). Dual‐luciferase reporter assay showed that compared to the negative control, miR‐874‐3p mimics significantly reduced the luciferase activity of wild‐type ATG16L1 3′‐UTR reporter vectors but not that of mutant 3′‐UTR reporter vectors (Figure 7b). miR‐874‐3p mimics also significantly attenuated, whereas its inhibitor enhanced the expression of ATG16L1 protein in THP‐1 macrophages regardless of the BCG infection (Figure 7c and d), suggesting that miR‐874‐3p can suppress the expression of ATG16L1 by binding to its 3′‐UTR. Moreover, the PBMCs of patients with active TB significantly decreased ATG16L1 mRNA levels as compared to those of healthy controls (Figure 7e). Similar results were also observed in H37Rv‐infected THP‐1 macrophages (Figure 1f). A correlation analysis revealed that ATG16L1 expression was significantly and positively correlated with circTRAPPC6B expression (Figure 7f) but negatively with miR‐874‐3p expression (Figure 7g). Western blot assay demonstrated that the silencing of circTRAPPC6B decreased the level of ATG16L1 protein in BCG‐infected macrophages (Figure 7h). Interestingly, the overexpression of circTRAPPC6B increased the expression of ATG16L1 protein (Figure 7i). Thus, the data demonstrated that circTRAPPC6B antagonises miR‐874‐3p to counter its suppression on ATG16L1 expression, thereby activating autophagy to eliminate Mtb in macrophages.

## Discussion

In this study, consistent with the previous findings,^22^ we demonstrated that circTRAPPC6B expression is significantly reduced in PBMCs of TB patients, BCG‐infected human peripheral monocytes, and BCG‐infected THP‐1 macrophages as compared to that in corresponding controls. Furthermore, our previous study demonstrated that the overexpression of circTRAPPC6B inhibits intracellular Mtb growth while activating autophagy in Mtb‐infected macrophages. In addition, circTRAPPC6B targets miR‐874‐3p and might abolish its suppression on ATG16L1, thereby activating autophagy to eliminate Mtb in macrophages.

CircRNAs are widely expressed in mammalian cells. The unique loop structure of circRNAs makes them resistant to RNase R degradation^24^ and are more stable biomarkers than linear RNAs.^25^ In the present study, bioinformatics analysis showed that circTRAPPC6B is derived from the back‐splicing of exons 3 and 4 of the TRAPPC6B gene. RNAse R digestion fails to reduce the production of circTRAPPC6B by PCR, hinting at a circTRAPPC6B that has a circular structure. In addition to the weak expression of circTRAPPC6B in PBMCs of TB patients, we further found that anti‐TB treatment can rescue its expression. Additionally, the AUC of circTRAPPC6B in active TB was 0.8609. These findings suggested that circTRAPPC6B may serve as a biomarker for diagnosing and monitoring treatment responses in TB.

The low expression of circTRAPPC6B in PBMCs, monocytes and macrophages with Mtb infection suggested an unfavorable role of circTRAPPC6B in Mtb growth. As expected, our data showed that the overexpression of circTRAPPC6B suppresses Mtb growth in BCG‐infected THP‐1 macrophages and H37Rv‐infected primary spleen macrophages in macaques. Because autophagy plays a vital role in eliminating intracellular Mtb,^11^ we hypothesised that the overexpression of circTRAPPC6B might activate autophagy to eliminate Mtb in macrophages. Intriguingly, the gain‐ and loss‐of‐function assays showed that the overexpression of circTRAPPC6B significantly enhances, whereas knockdown of circTRAPPC6B attenuates LC3‐II expression in THP‐1 macrophages regardless of BCG and H37Rv infection, suggesting that circTRAPPC6B activates autophagy in macrophages. Although we could not establish a direct link between autophagy activation and Mtb elimination in circTRAPPC6B‐overexpressing macrophages, accumulating evidence suggested that the stimulation of autophagy suppresses, whereas the impairment increases Mtb survival in macrophages.^10^, ^11^ Therefore, we speculated that circTRAPPC6B suppresses Mtb growth in macrophages by activating autophagy, serving as a potential therapeutic agent in TB treatment.

Considering the well‐established interaction between circRNAs and miRNAs, we further identified the potential miRNA targets of circTRAPPC6B to investigate the mechanisms underlying its role in Mtb growth and autophagy in macrophages. Bioinformatics analysis demonstrated that circTRAPPC6B harbours a miR‐874‐3p binding site. Dual‐luciferase reporter assay, RNA FISH and RNA pull‐down further confirmed that circTRAPPC6B acts as a miR‐874‐3p sponge in macrophages. These findings suggested that circTRAPPC6B and miR‐874‐3p exhibit opposite expression patterns in macrophages. As expected, both PBMCs of patients with active TB and THP‐1 macrophages with Mtb infection show significantly increased miR‐874‐3p level compared to the corresponding controls, contrary to that observed in circTRAPPC6B expression. Moreover, circTRAPPC6B and miR‐874‐3p expression is significantly and negatively correlated in PBMC samples. These findings prompt us to explore further whether miR‐874‐3p plays opposite roles to circTRAPPC6B in Mtb growth and autophagy.

MiR‐874‐3p functions as a tumor suppressor in human cancers^26^; however, its role in Mtb growth and macrophage autophagy remains unknown. A recent study reported that miR‐874‐3p inhibits autophagy in HeLa cells.^23^ Consistently, our results showed that miR‐874‐3p mimics dramatically suppresses, whereas miR‐874‐3p inhibitor facilitates the conversion of LC3‐I to LC3‐II in THP‐1 macrophages regardless of BCG and H37Rv infection, suggesting that miR‐874‐3p plays an essential role in suppressing autophagy in macrophages. In addition, miR‐874‐3p is also essential for Mtb growth in macrophages, as evident from suppressed intracellular H37Rv growth in both THP‐1 and macaque spleen macrophages exposed to miR‐874‐3p inhibitor. Furthermore, miR‐874‐3p can block the inductive role of circTRAPPC6B in macrophage autophagy during Mtb infection. To the best of our knowledge, this is the first study reporting the essential roles of miR‐874‐3p and its interaction with circTRAPPC6B in suppressing autophagy while promoting Mtb growth in macrophages.

Reportedly, miR‐874 targets ATG16L1 in gastric cancer cells and HeLa cells.^23^, ^27^ Consistently, our results showed that miR‐874‐3p suppresses ATG16L1 expression by binding to its 3′‐UTR. In addition, PBMCs of active TB patients have significantly decreased ATG16L1 mRNA levels compared to those of healthy controls. The mRNA expression of ATG16L1 is significantly and positively correlated with that of circTRAPPC6B but negatively correlated with that of miR‐874‐3p in PBMCs. Therefore, we speculated that circTRAPPC6B antagonises miR‐874‐3p to counter its suppression of ATG16L1 expression. ATG16L1, ATG5 and ATG12 form a large protein complex (the ATG16L1 complex) that is essential for autophagosome formation.^28^ ATG16L1 is involved in host immune responses against intracellular bacteria and viruses via autophagy.^29^ siRNA targeted to ATG16L1 impairs autophagy in macrophages infected with adherent‐invasive *Escherichia coli*, in turn, increasing the proinflammatory cytokine secretion and intracellular bacterial population.^30^ In addition, knock‐in mice harbouring a missense ATG16L1 variant showed a defective clearance of the ileal pathogen *Yersinia enterocolitica* and elevated inflammatory responses due to diminished autophagy.^31^ Based on these findings, we speculated that circTRAPPC6B overexpression might abrogate the suppression of miR‐874‐3p on ATG16L1 expression, thereby activating autophagy to eliminate Mtb in macrophages. However, we did not establish a direct causative correlation between the expression of circTRAPPC6B and ATG16L1, which should be addressed in future studies.

**Figure 8:**
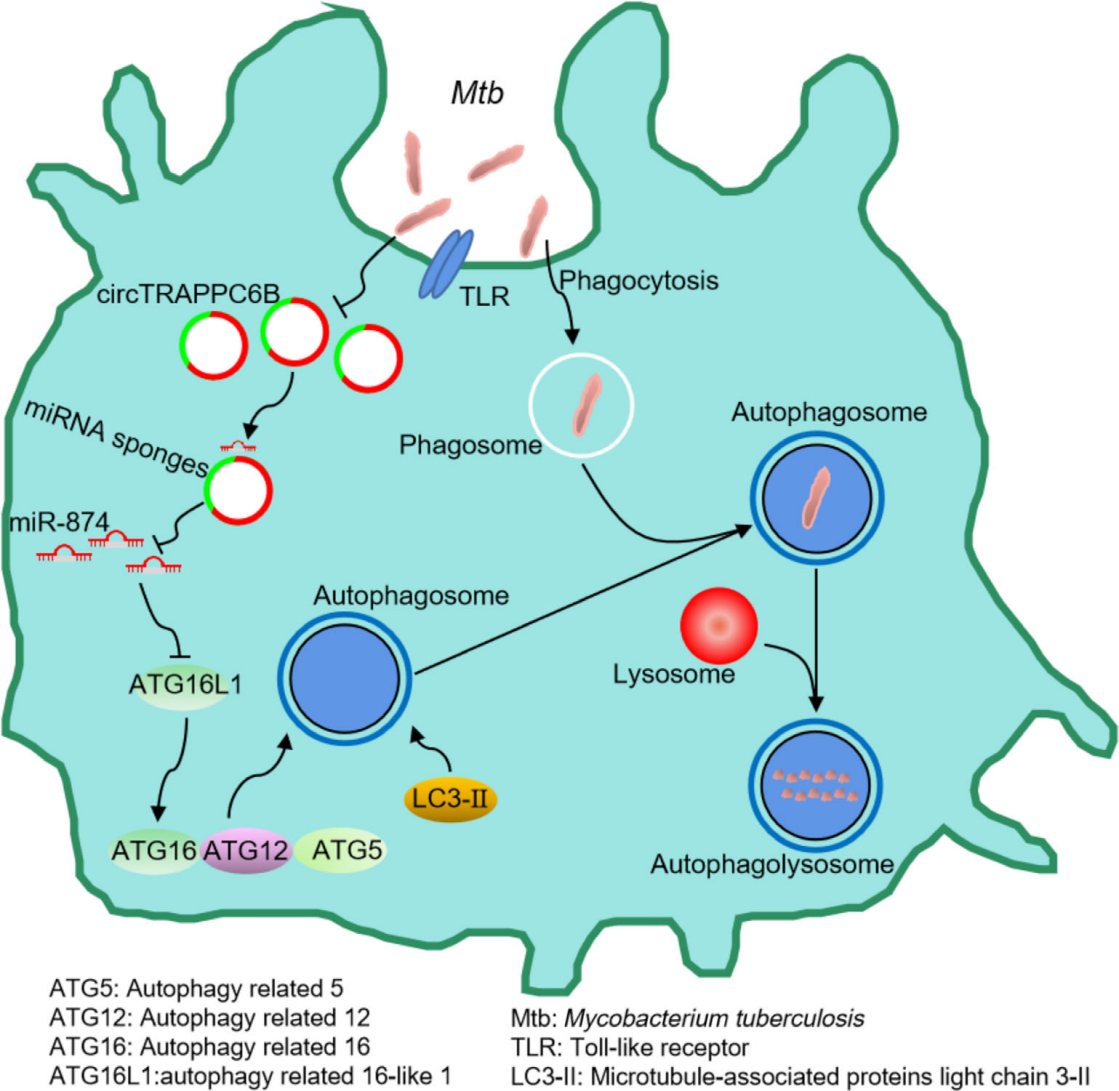
Schematic diagram of the circTRAPPC6B/miR‐874‐3p/ATG16L1 axis.

In summary, we demonstrated that circTRAPPC6B inhibits intracellular Mtb growth while inducing autophagy in Mtb‐infected macrophages. CircTRAPPC6B acts as a novel competitive endogenous RNA to sponge miR‐874‐3p in macrophages, suggesting that circTRAPPC6B abolishes the suppression of miR‐874‐3p on ATG16L1 expression to regulate Mtb growth and autophagy in macrophages (Figure 8). This study provides circTRAPPC6B as a potential therapeutic agent for TB control.

## Methods

### Ethics statement

The protocol of using human blood samples *in vitro* was approved by the Institutional Review Boards for Human Donors’ Research and Institutional Biosafety Committee at Institute Pasteur of Shanghai, Guangdong Medical University, and Dongguan 6th Hospital (Dongguan, China). The protocols of using macaque spleen tissue samples and *Mycobacterium* H37Rv strain were approved by the Institutional Review Boards for Biosafety Committee at the University of Illinois, Chicago College of Medicine (IL, USA). All studies complied with the guidelines of the Office for Human Research Protection. All patients and healthy controls provided written informed consent before initiation of the study.

### Patients and cell samples collection

A total of 32 patients (17–70‐year‐old) with active pulmonary TB admitted to Dongguan 6^th^ Hospital were recruited in this study. The diagnosis was made, as described previously.^32^ According to the standard methods, the sputum samples of all were tested by Ziehl–Neelsen acid‐fast staining and Lowenstein–Jensen slant culture. A total of 31 healthy volunteers (20–65 years of age) without bacteriological and clinical evidence of TB were enrolled as controls (Supplementary table 1). Patients with active TB received individualised anti‐TB treatments, including isoniazid, rifampicin, pyrazinamide and ethambutol.

### Cell lines and *Mycobacterium*

The human THP‐1 monocyte cell line was grown in RPMI 1640 supplemented with L‐glutamine (2 mm), sodium pyruvate (1 mm) and 10% heat‐inactivated foetal bovine serum (FBS; Life Technologies, Carlsbad, CA, USA). Before mycobacterial infection, THP‐1 cells were treated with 50 ng mL^−1^ phorbol 12‐myristate 13‐acetate (PMA; Sigma‐Aldrich, St. Louis, MO, USA) for 48 h to allow differentiation into macrophages. The cells were then washed three times with prewarmed phosphate‐buffered saline (PBS) and maintained in antibiotic‐free media at 37°C in a humidified atmosphere of 5% CO_2_ for subsequent use. All the cell lines were tested for *Mycoplasma* infection using a 16S‐based PCR. New cultures were established monthly from frozen stocks.

Macaque spleen macrophages were enriched from macaque spleen tissues. The spleen macrophages were grown in RPMI 1640 supplemented with L‐glutamine (2 mm), sodium pyruvate (1 mm) and 10% heat‐inactivated FBS for 7 days.

Human PBMCs were prepared, as described previously.^32^, ^33^ Peripheral blood samples (10 mL) were collected in EDTA‐tubes from TB patients and healthy volunteers and separated by density gradient centrifugation using Ficoll‐Paque PLUS medium (GE Healthcare, Chicago, IL, USA). Human monocytes were sorted from PBMCs by immunomagnetic positive selection (STEMCELL Technologies, Vancouver, BC, Canada).

*Mycobacterium* BCG, GFP‐BCG and the H37Rv strains were grown in Difco Middlebrook 7H9 broth supplemented with 10% oleic acid‐albumin‐dextrose‐catalase (OADC; BD Biosciences, San Jose, CA, USA), 0.05% (v/v) Tween 80 and 0.2% (v/v) glycerol at 37°C. Subsequently, the cells were infected with BCG or GFP‐BCG at an MOI 10 overnight and H37Rv at an MOI 1 for 4 h.

### Oligonucleotide and plasmid transfection

The THP‐1 monocytes were seeded in a 12‐well or 24‐well plate and incubated at 37°C overnight. Before mycobacterial infection, THP‐1 cells were treated with PMA (Sigma‐Aldrich) for 48 h to differentiate into macrophages. circTRAPPC6B siRNA, miR‐874‐3p mimics, or miR‐874‐3p inhibitors (Ribobio, Guangzhou, China) were transfected using Lipofectamine 2000 (Life Technologies) according to the manufacturer’s protocol.

CircTRAPPC6B cDNA was synthesised and cloned into a pHBAd‐EF1‐CircRNA‐CMV‐GFP vector (Hanbio Biotechnology, Shanghai, China), followed by transfection into cells using Lipofectamine 3000 (Life Technologies) according to the manufacturer’s protocol.

### RNA preparation, RNAse R treatment and PCR

Total RNAs were isolated using TRIzol reagent (Invitrogen, Carlsbad, CA, USA) according to the manufacturer’s protocol. An equivalent of 2 μg total RNA was incubated with 2 U RNAse R (Epicenter Technologies, San Diego, CA, USA) in 1× RNase R reaction buffer for 10 min at 37°C, followed by reverse transcription into cDNA using PrimeScript RT Reagent (TaKaRa, Japan). Subsequently, qRT‐PCR was performed using a TransStart Tip Green qPCR SuperMix (Transgen, Beijing, China) on a QuantStudio™ 6 Flex Real‐Time PCR System (Applied Biosystems, Foster City, CA, USA). GAPDH and *RNU6‐1* were used as internal controls for circRNA, mRNA and miRNA, respectively. All qRT‐PCR reactions were performed in triplicate. The data were analysed using the $2^{‐\Delta \Delta C_{t}}$ method to calculate the relative expression of the target gene. PCR primers are listed in Supplementary table 2.

### Western blot analysis

Proteins were isolated from THP‐1 cells using a protein extraction kit (Beyotime, Shanghai, China) according to the manufacturer’s protocol. The protein concentration was measured using a BCA kit (Beyotime). An equivalent of protein samples was separated using SDS‐PAGE and then transferred onto a PVDF membrane (Millipore, Burlington, MA, USA). After blocking with 5% skim milk in TBST buffer for 2 h, the membrane was probed at 4°C overnight with monoclonal antibody against LC‐3 (1: 1000, Proteintech, Rosemont, IL, USA) or *ATG16L1* (1: 1000, Proteintech). Following 4 × 5 min washes with TBST, the membrane was incubated with a corresponding HRP‐labelled secondary antibody for 2 h at room temperature. Subsequently, the immunoreactive signals were visualised using an enhanced chemiluminescence detection system with a chemiluminescence substrate (Thermo Scientific, Waltham, MA, USA). β‐Actin (Beyotime, Shanghai, China) was used as an internal control.

### *Mycobacteria* CFU assay

Transfected THP‐1 and macaque spleen macrophages were infected with H37Rv at an MOI of 1. At 4 h after infection, extracellular non‐internalised bacilli were removed by washing three times with prewarmed PBS, and this time point was recorded as 0. Then, the supernatant of the cells was removed, and the infected cells were lysed in 1 mL sterile water containing 0.03% SDS for 20 min. A 10‐fold serial dilution was performed for quantitative culturing. Aliquots (100 mL) were plated in triplicate on Middlebrook 7H11 agar plates supplemented with 10% OADC enrichment for 2–3 weeks until colonies were sufficiently large for counting. The viability of *Mycobacteria* was quantified by counting CFU.

### Bioinformatics prediction and luciferase assay

The potential miRNA targets of circTRAPPC6B were predicted using CircInteractome (https://circinteractome.nia.nih.gov), miRDB (http://www.mirdb.org/) and RegRNA 2.0 (regrna2.mbc.nctu.edu.tw) databases. The potential targets of miR‐874 were predicted using TargetScan (http://www.targetscan.org), miRanda (http://www.microrna.org/microrna/home.do) and miRDB (http://www.mirdb.org/) databases. circTRAPPC6B/ATG16L1 with wild‐type or mutant miR‐874 binding site was constructed and subcloned into the psiCHECK2 vector (Promega, Madison, WI, USA). HEK293T cells were cotransfected with the reporter plasmids and miR‐874 mimics or negative control mimics using Lipofectamine 2000. The Firefly luciferase and Renilla luciferase signals were detected using a Dual‐Luciferase Reporter System Kit (Promega).

### FISH

To detect the intracellular location of circTRAPPC6B and miR‐874 as well as the colocalisation of circTRAPPC6B and miR‐874, RNA FISH was performed according to the protocol of Guangzhou Geneseed Technology (Guangzhou, China). The biotin‐conjugated circTRAPPC6B and digoxin‐conjugated miR‐874‐3p probes were synthesised by Guangzhou Geneseed Biotechnology. Briefly, THP‐1 cells were rinsed in PBS and fixed in 4% formaldehyde with RNase‐free PBS for 5 min at room temperature. Then, the cells were incubated with absolute ethyl alcohol for 1 min. circTRAPPC6B and miR‐874‐3p probes were hybridised with the samples in the dark at 37°C overnight. The cells were blocked with 3% bovine serum albumin (BSA) for 30 min and incubated with anti‐biotin CY3‐conjugated or anti‐digoxin FITC‐conjugated secondary antibody for 1 h at 37°C. The cells were visualised using laser‐scanning confocal microscopy (Leica TCS SP2 AOBS, Germany).

### CircRNA pull‐down assay

All the following experiments were performed at room temperature except for those mentioned otherwise. The probes are listed in Supplementary table 2. The cells were lysed using lysis buffer containing Superase‐In and protease inhibitors for Western blot and IP (P0013, Beyotime, Shanghai, China).

An equivalent of 1 × 10^7^ THP‐1 cells was fixed with 1% formaldehyde for 10 min, lysed for 10 min at 4°C, and sonicated, followed by centrifugation at 10 000 *g* for 10 min. A volume of 50 μL supernatant was used for input analysis. The remaining volume was incubated with 3 μg circTRAPPC6B specific probes or control probes at 30°C for 1 h, rotating at 10 rpm. The streptavidin magnetic beads (Life Technologies) were washed twice with 500 μL lysis buffer and resuspended in 1000 μL lysis buffer. The biotin‐coupled RNA complex was pulled down by incubating the cell lysates with 50 μL beads for 3 h on the rotator at 10 rpm, followed by RNA extraction using TRIzol and qRT‐PCR detection.

### MiRNA pull‐down assay

Approximately 2 × 10^6^ cells were transfected with 50 μm of biotinylated‐miRNA mimics or nonsense control (GenePharma, Shanghai, China) at 50% confluency. At 24 h after transfection, the cells were harvested, washed in PBS, and lysed with lysis buffer. A volume of 50 μL of washed streptavidin magnetic beads was blocked for 2 h and then added to each reaction tube to pull down the biotin‐coupled RNA complex. Subsequently, the tubes were incubated for 4 h on the rotator at a low speed of 10 rpm, and then, washed with lysis buffer five times. TRIzol LS (Life Technology, USA) was used to recover RNAs, specifically interacting with miRNA. The abundance of circTRAPPC6B in the bound fractions was evaluated by qRT‐PCR and agarose gel electrophoresis.

### Immunofluorescence analysis

THP‐1 cells were seeded on a Nunc glass‐bottom dish (Thermo Fisher Scientific) and incubated in RPMI 1640 medium at 37°C in a humidified atmosphere with 5% CO_2_ overnight. The cells were washed with PBS three times after corresponding treatment, fixed with 4% paraformaldehyde for 15 min, and permeabilised with 0.25% Triton X‐100 for 10 min. After blocking with 5% skim milk in TBST for 30 min, the cells were incubated with anti‐LC3B (Cell Signal Technology, Danvers, MA, USA) for 2 h at room temperature, followed by incubation with Alexa Fluor^®^ 594‐conjugated anti‐Rabbit IgG (Thermo Fisher Scientific) for 1 h at room temperature. The nuclei were stained with DAPI for 5 min. After mounting, fluorescence images were acquired using a confocal laser‐scanning microscope (Zeiss, Jena, Germany). The experiments were repeated three times. A total of 100 cells were selected to calculate the average value at each step, and the average obtained from three independent experiments was plotted.

### Statistical analysis

All data were analysed using SPSS17.0 (IBM, Armonk, NY, USA) and plotted using GraphPad Prism 5 software (GraphPad, USA). Data were expressed as mean ± standard error of the mean (SEM). The Student’s *t*‐test and the paired *t*‐test were used to assess the statistical significance between the two groups. ANOVA with *Dunnett’s* multiple comparisons test was used to compare more than two groups. *Pearson’s* analysis was used to analyse the correlation between the two groups. A *P*‐value < 0.05 was considered to be statistically significant.

## Conflict of interest

The authors declare no conflict of interest.

## Author contributions

**Hou‐Long Luo:** Conceptualization; Data curation; Formal analysis; Investigation; Methodology; Writing‐original draft. **Jiang Pi:** Conceptualization; Data curation; Formal analysis; Investigation; Methodology; Writing‐original draft. **Jun‐Ai Zhang:** Conceptualization; Data curation; Formal analysis; Investigation; Methodology; Writing‐original draft. **En‐Zhuo Yang:** Data curation; Formal analysis; Writing‐review & editing. **Huan Xu:** Data curation; Formal analysis; Writing‐review & editing. **Hong Luo:** Data curation; Formal analysis; Writing‐review & editing. **Ling Shen:** Data curation; Formal analysis; Writing‐review & editing. **Ying Peng:** Data curation; Formal analysis; Writing‐review & editing. **Gan‐Bin Liu:** Data curation; Formal analysis; Writing‐review & editing. **Cai‐Mei Song:** Data curation; Formal analysis; Writing‐review & editing. **Ke‐Yue Li:** Data curation; Formal analysis; Writing‐review & editing. **Xian‐Jin Wu:** Data curation; Formal analysis; Writing‐review & editing. **Bi‐Ying Zheng:** Data curation; Formal analysis; Writing‐review & editing. **Hong‐Bo Shen:** Data curation; Formal analysis; Writing‐review & editing. **Zheng Chen:** Conceptualization; Formal analysis; Funding acquisition; Project administration; Writing‐review & editing. **Jun‐Fa Xu:** Conceptualization; Formal analysis; Funding acquisition; Project administration; Writing‐review & editing.
