# Supplementary material for: Circular RNA TRAPPC6B inhibits intracellular Mycobacterium tuberculosis growth while inducing autophagy in macrophages by targeting microRNA‐874‐3p
Source: Clin Transl Immunology. 2021 Feb 18;10(2):e1254. doi: 10.1002/cti2.1254 (PMC7890665; doi:10.1002/cti2.1254)
Supplement: Supplementary file 1 [file CTI2-10-e1254-s001.doc]

**Supplementary tables**

**Supplementary table 1.** The clinical data of studied subjects

| Groups | APTB (n = 32) | HC (n = 31) |
| --- | --- | --- |
| Age (years) | (17~70) | (20~65) |
| Age (Mean ± SEM) | 40.09 ± 13.76 | 38.64 ± 11.70 |
| Gender (Male/Female) | 22/10 | 20/11 |
| Newly/Relapsed | 27/5 | -/- |
| Sputum smear (+/-) | 24/8 | -/- |

Newly, Newly diagnosed patients; Relapsed, Relapsed patients. There were no significant differences among age and gender groups (*P* > 0.05).

**Supplementary table 2.** Primers and probes used for RT-qPCR, circRIP and FISH

| Primers/Probes | Sequence (5’ to 3’) |
| --- | --- |
| Ad-hsa_circ_0005836-Kpn/Kpn-F | tactaatgactttttttttatacttcagGTTTACAAAAGATACTGCAA |
| Ad-hsa_circ_0005836-Kpn/Kpn-R | agcctaattcttttccttgcttcttacCTTAGATGCATGTTCTAAAT |
| hsa_circ_0005836-F1 | GGACTACGGTATTCAAGAAA |
| hsa_circ_0005836-R1 | GTTGTCCTGAAGTACATAGA |
| hsa_circ_0005836-F2 | GTTAGATATCATGAAGTTCA |
| hsa_circ_0005836-R2 | CCTGCAGACATCTGAGTAAG |
| GAPDH-F | GAAGGTCGGAGTCAACGGATT |
| GAPDH-R | CCTGGAAGATGGTGATGGGATT |
| hsa_circ_0005836-F | ACTCATCCTTGAACCTTGC |
| hsa_circ_0005836-R | GGCATCTATGTACTTCAGGAC |
| ATG16L1-F | CAAGCCGAATCTGGACTGTG |
| ATG16L1-R | CCAGAGTTTGAGAGTCCGGT |
| TRAPPC6B-F | GATGAGGCGTTGTTTTTGCTT |
| TRAPPC6B-R | CCCACTCGAAACCCCATGTT |
| circTrappc6b-F | TGAGGACAAATCATCAGGGCA |
| circTrappc6b-R | TCGTAAACCTTGGACGCATGT |
| hsa_circ_0005836-Biotin (FISH probe) | CAGTATCTTTTGTAAACCTTAGATGCA |
| hsa-miR-874-3p-Digoxin (FISH probe) | TCGGTCCCTCGGGCCAGGGCAG |
| NC probe (RNA pull down) | 5’biotin-GUGUAACACGUCUAUACGCCCA |
| hsa_circ_0005836 probe (RNA pull down) | 5’biotin-UGCAGUAUCUUUUGUAAACCUUAGAUGCAUGUUCUAAAU |
| hsa-miR-874-3p probe (RNA pull down) | 5’biotin-UCGGUCCCUCGGGCCAGGGCAG |

**Supplementary table 3.** Antibody used in western blotting and IF

| Name | Catalog number | Company |
| --- | --- | --- |
| β-actin Antibody | AA128 | Beyotime, Shanghai, China |
| LC3 Antibody | 14600-1-AP | Proteintech, USA |
| ATG16L1 Antibody | 19812-1-AP | Proteintech, USA |
| Anti-rabbit IgG (H+L), F(ab')2 Fragment (Alexa Fluor® 594 Conjugate) | 8889 | Cell Signaling Technology, USA |
| LC3B Antibody | 2775 | Cell Signaling Technology, USA |

**Supplementary figures**


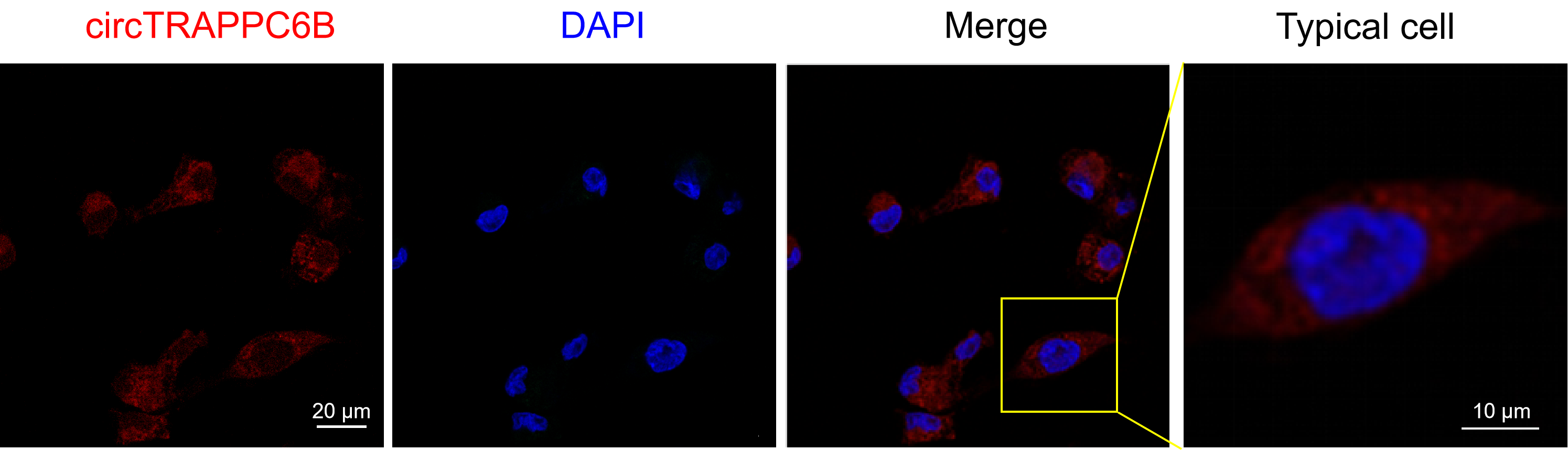


**Supplementary figure 1.** Enlarged image of Figure 2d. **(d)** After infection with *BCG* at MOI = 10 for 24 h, THP-1 macrophages were collected and incubated with biotin-conjugated *circTRAPPC6B* probe overnight at 37℃. Representative immunofluorescence confocal image showing the subcellular distribution of *circTRAPPC6B*. Scale bar, 20 μm. Scale bar of typical cell, 10 μm.


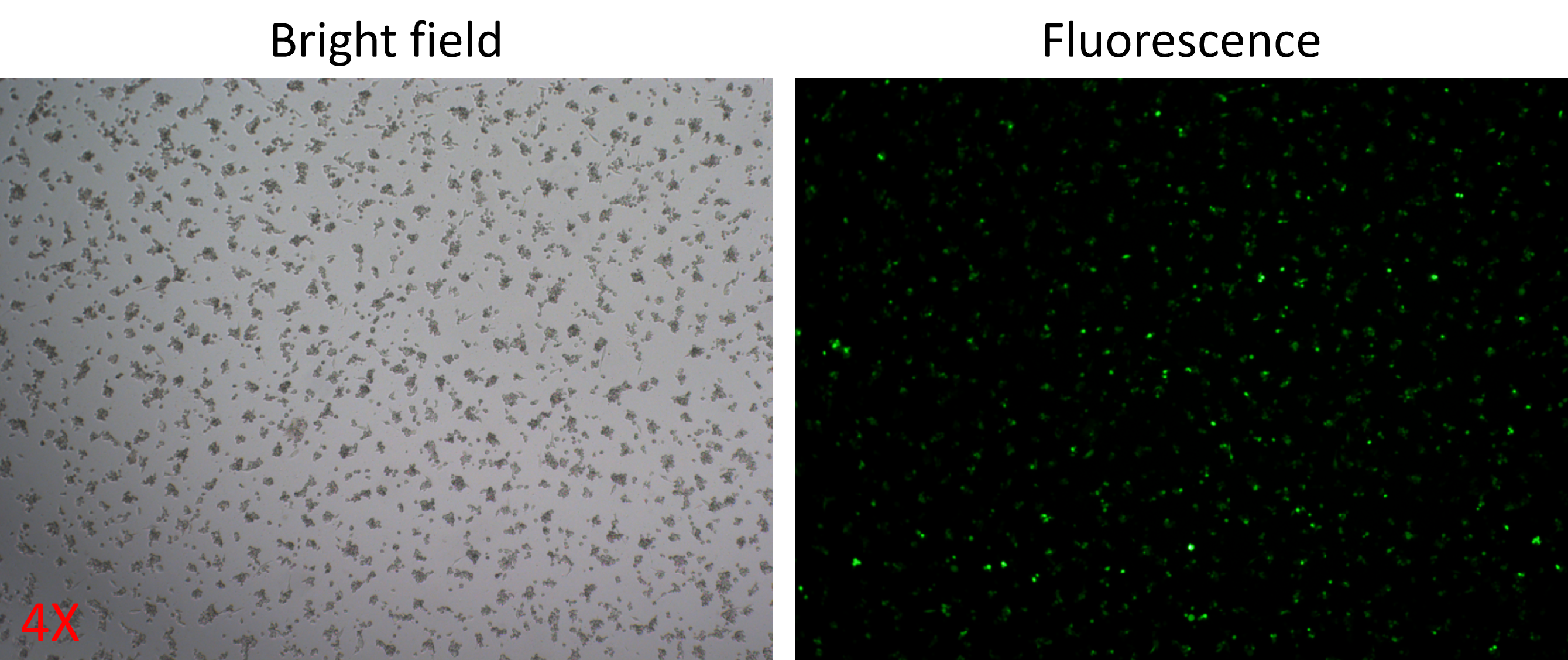


**Supplementary figure 2.** After transfection with *circTRAPPC6B*-overexpressing vectors (pHBAd-cir) in THP-1 macrophages for 24 h, representive fluorescence microscope image showing the transfection efficiency of *circTRAPPC6B*.


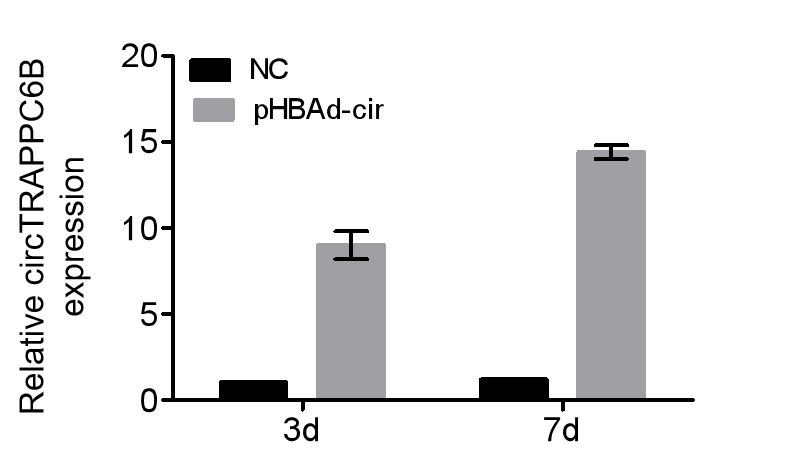


**Supplementary figure 3.** qRT-PCR was performed to assay the expression efficiency of pHBAd-*circTRAPPC6B* after being transfected for 3 days or 7 days.


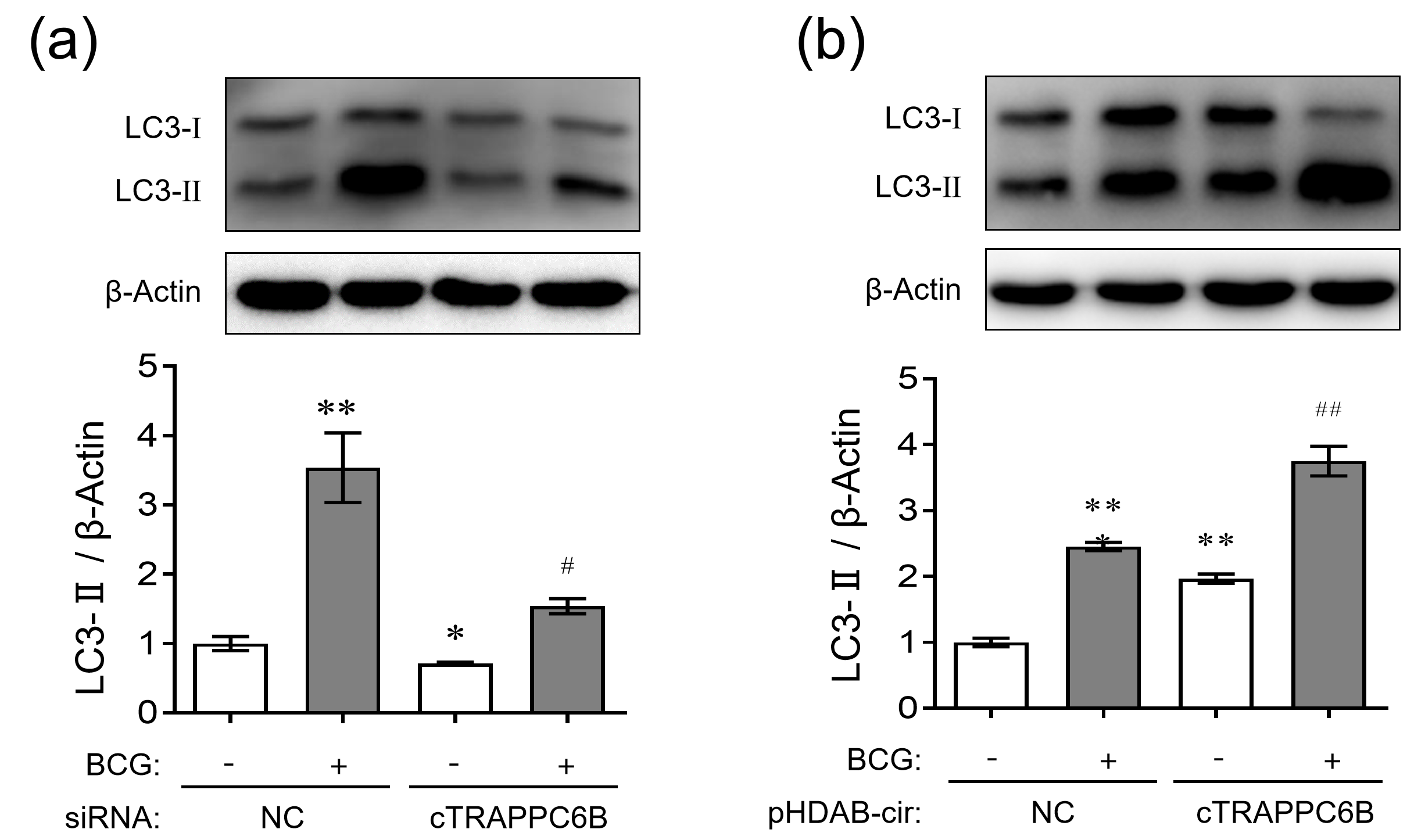


**Supplementary figure 4.** **(a, b)** THP-1 macrophages were transfected with siRNA against *circTRAPPC6B* **(a)** or plasmids overexpressing *circTRAPPC6B* **(b)** prior to *BCG* infection. Western blot analysis was performed to determine the protein expression of LC3-I and LC3-II. The data were obtained from three independent experiments. Data are expressed as *mean* ± *SEM*. ^*^*P* < 0.05, ^**^*P* < 0.01 *vs*. uninfected and untransfected control. ^#^*P* < 0.01, ^##^*P* < 0.01 *vs*. *BCG*-infected but untransfected control.


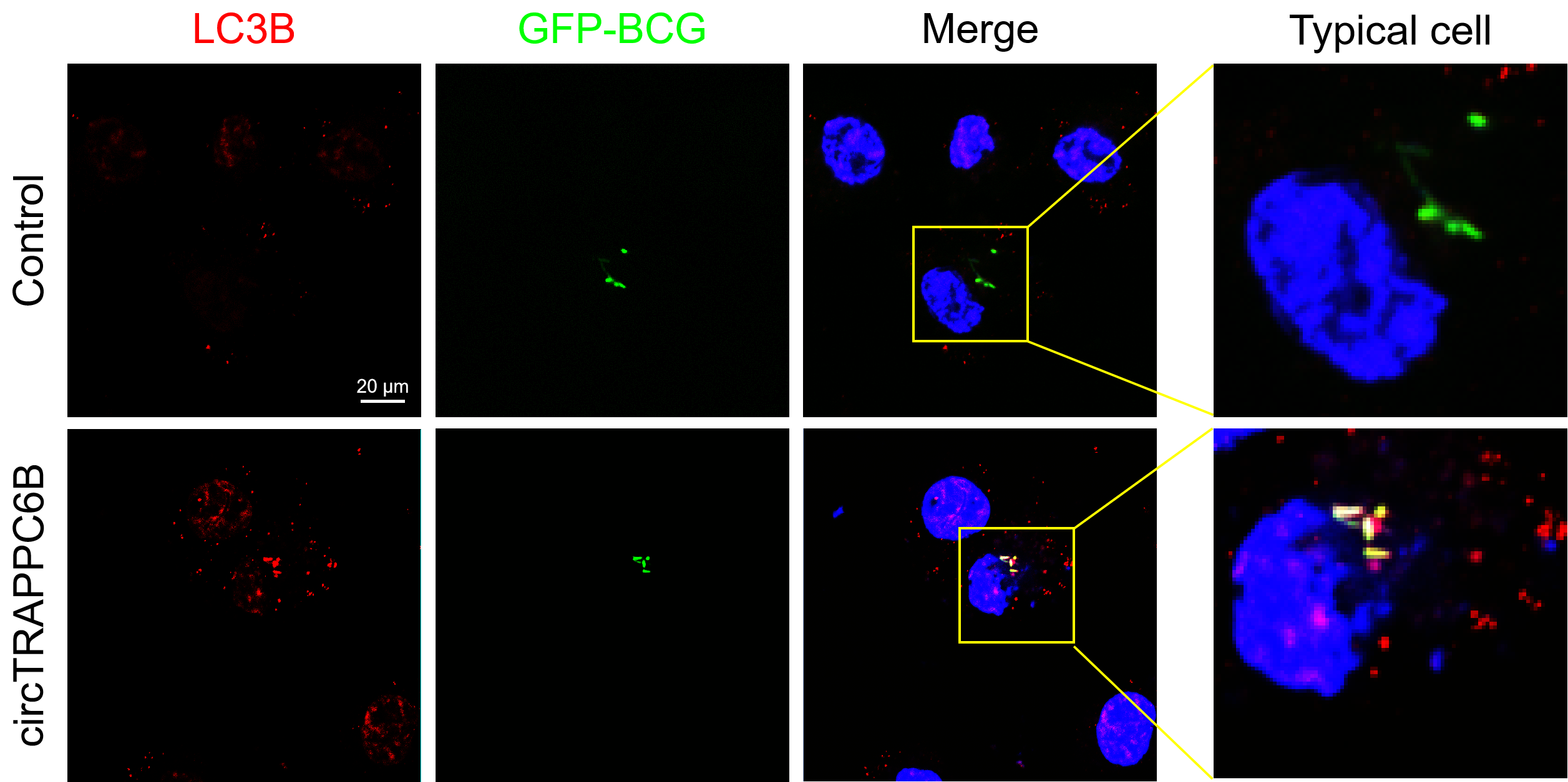


**Supplementary figure 5.** Enlarged image of Figure 3e. After transfection with *circTRAPPC6B* overexpressing vectors or control vectors (NC) for 24h, THP-1 macrophages were infected with *GFP-BCG* at MOI = 10 for 24 h. THP-1 macrophages were incubated with anti-LC3 for 2h at room temperature, then fluorescently labeled secondary Ab for 1 h at room temperature. Representative immunofluorescence confocal image of THP-1 macrophages showing the change of LC3B puncta and *GFP-BCG* colocalization. Scale bar, 20 μm.


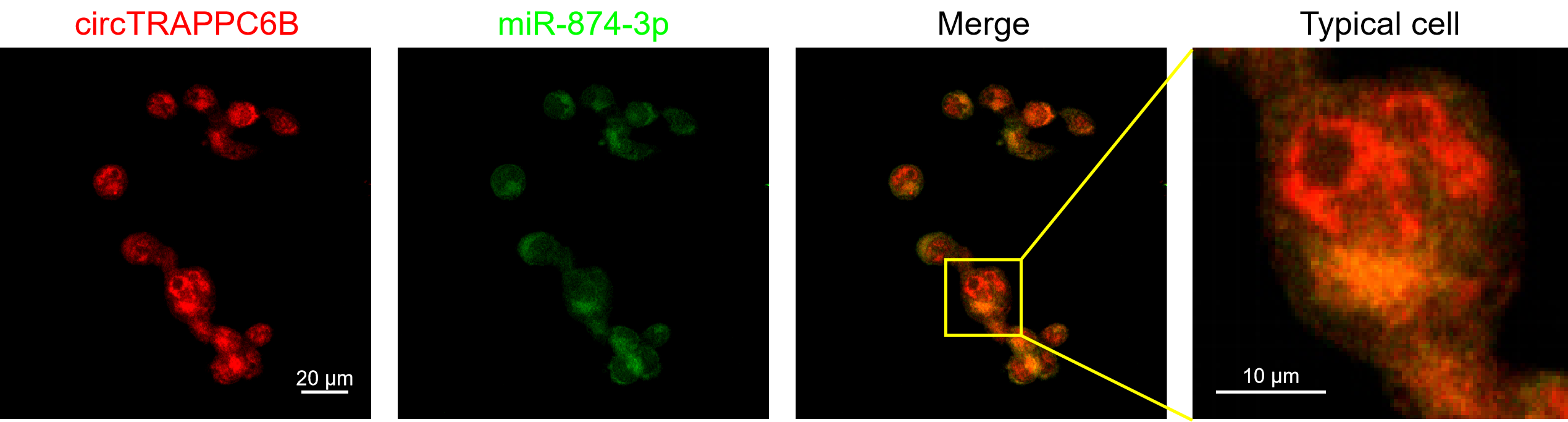


**Supplementary figure 6** Enlarged image of Figure 4c. After infection with *BCG* at MOI = 10 for 24 h, THP-1 macrophages were collected and incubated with biotin-conjugated *circTRAPPC6B* and digoxin-conjugated miR-874 probes overnight at 37℃. Representative immunofluorescence confocal image showing partly colocalization of *circTRAPPC6B* with *miR-874-3p* in the cytoplasm of THP-1 macrophages. Scale bar, 20 μm. Scale bar of typical cell, 10 μm.


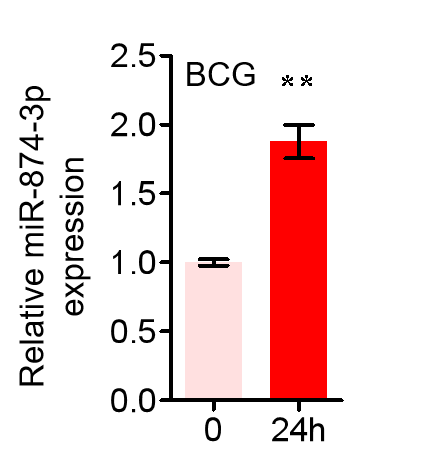


**Supplementary figure 7.** qRT-PCR analysis of *miR-874-3p* expression in *BCG*-infected THP-1 macrophages for 24 h. The data were obtained from three independent experiments. Data are expressed as *mean* ± *SEM*. ^**^*P* < 0.01 *vs*. 0 d.


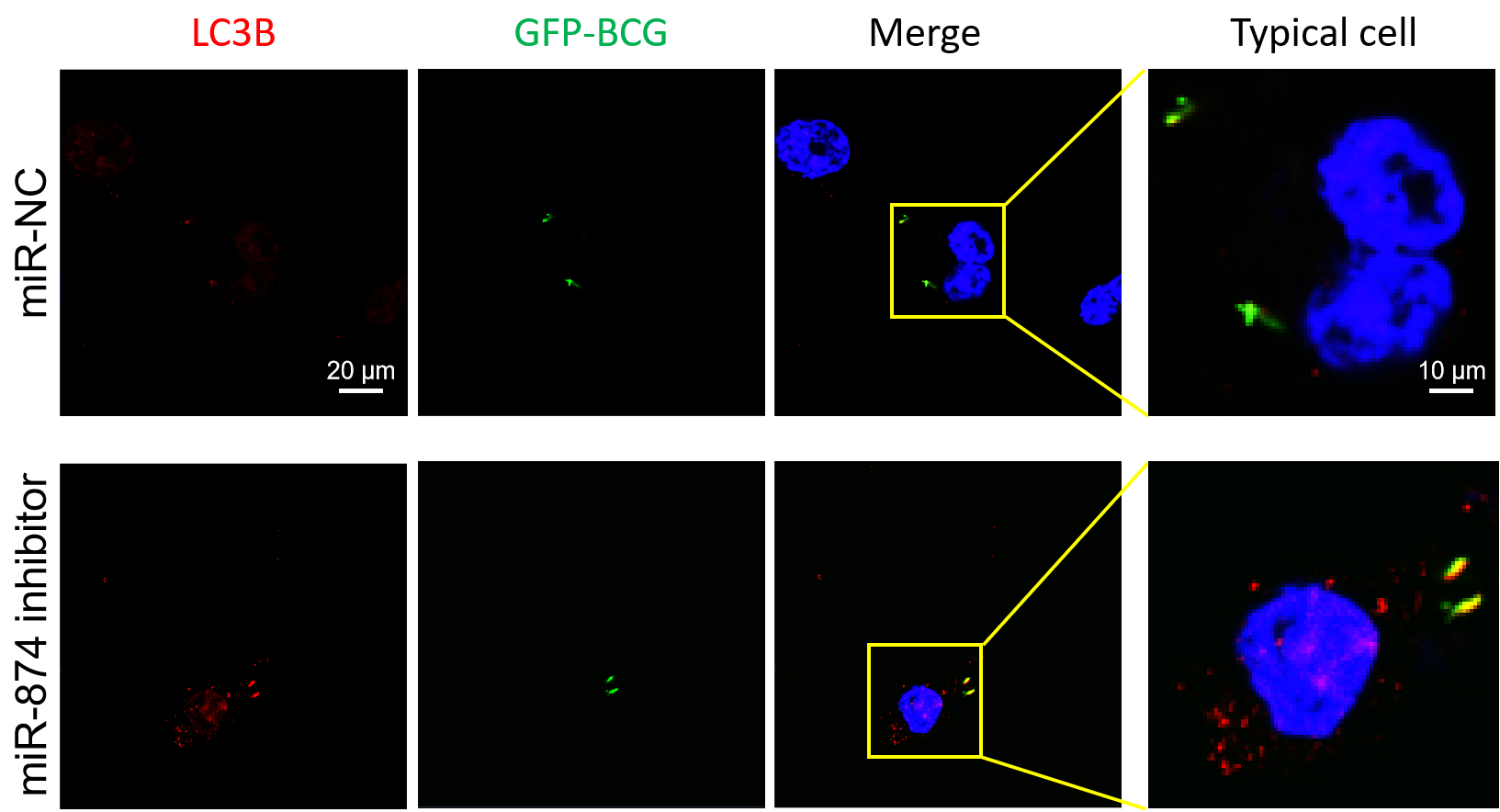


**Supplementary figure 8** Enlarged image of Figure 5f. After transfection with *miR-874-3p* inhibitor or negative control (miR-NC) for 24h, THP-1 macrophages were infected with *GFP-BCG* at MOI = 10 for 24h. Then, THP-1 macrophages were incubated with anti-LC3B for 2h at room temperature, then fluorescently labeled secondary Ab for 1h at room temperature. representative immunofluorescence confocal image of THP-1 macrophages showing the change of LC3B puncta and *GFP-BCG* colocalization. Scale bar, 20 μm.


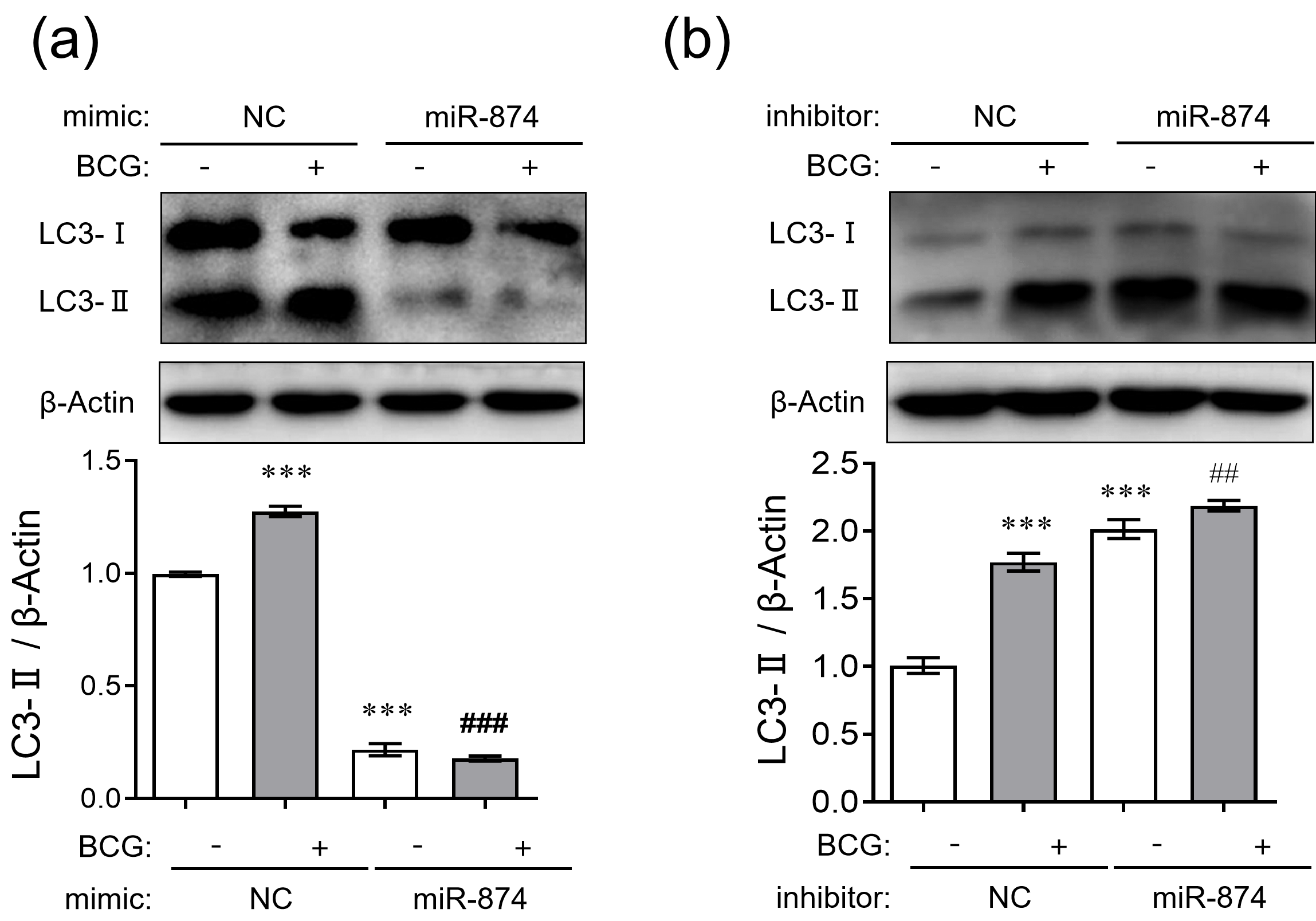


**Supplementary figure 9** **(a-b)** THP-1 macrophages were transfected with *miR-874-3p* mimics **(a)** or inhibitor **(b)** prior to *BCG* infection. Western blot analysis was performed to determine the protein expression of LC3-I and LC3-II. The data were obtained from three independent experiments. Data are expressed as *mean* ± *SEM*. ^***^*P* < 0.001 *vs*. uninfected and untransfected control; ^##^*P* < 0.01, ^###^*P* < 0.001 *vs*. *BCG*-infected but untransfected cells.


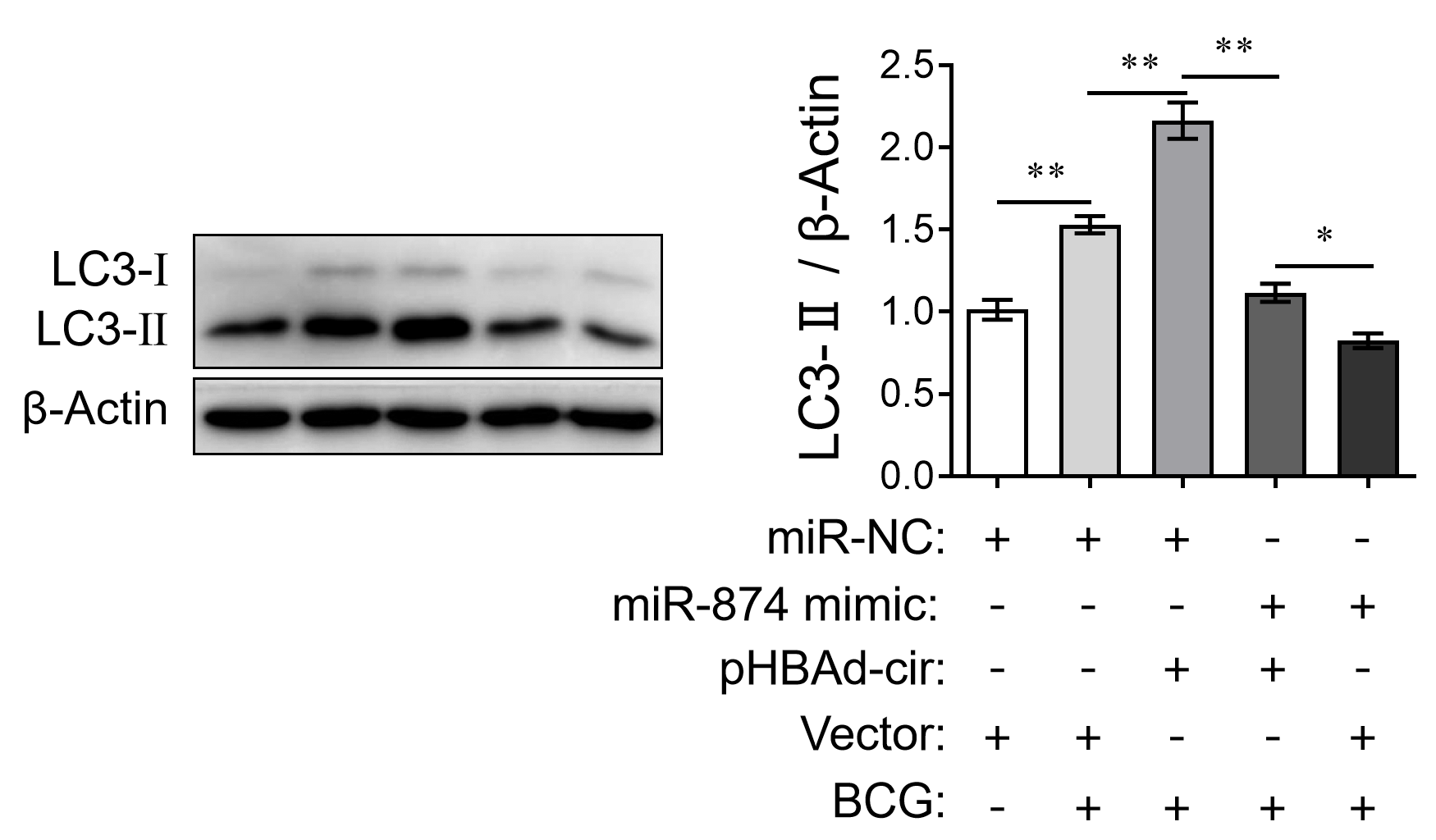


**Supplementary figure 10** THP-1 macrophages were transfected with *circTRAPPC6B*-overexpressing vectors (pHBAd-cir), *miR-874* mimics, or corresponding negative controls as indicated, followed by *BCG* infection. Western blot analysis determined the protein expression of LC3-I and LC3-II. The data were obtained from three independent experiments. Data are expressed as *mean* ± *SEM*. ^*^*P* < 0.05, ^**^*P* < 0.01.


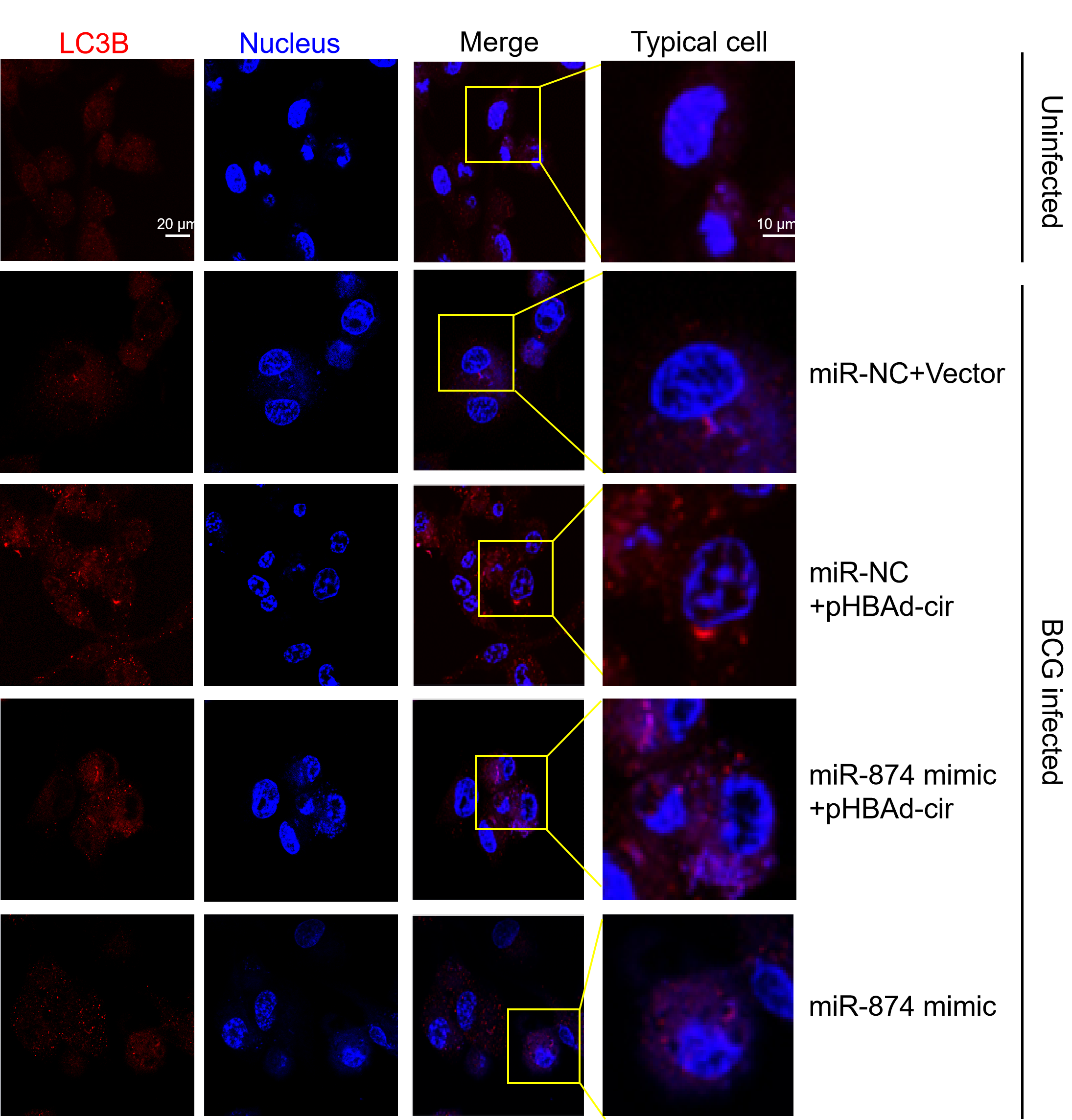


**Supplementary figure 11** Enlarged image of Figure 6b. THP-1 macrophages were incubated with anti-LC3B for 2h at room temperature, then fluorescently labeled secondary Ab for 1h at room temperature. representative immunofluorescence confocal image of *BCG*-infected THP-1 macrophages showing the change of LC3B puncta.
